# Gut Microbiota‐Derived Hyocholic Acid Enhances Type 3 Immunity and Protects Against *Salmonella enterica* Serovar Typhimurium in Neonatal Rats

**DOI:** 10.1002/advs.202412071

**Published:** 2024-12-31

**Authors:** Zhipeng Yang, Zhiyuan Lin, Yaojie You, Mei Zhang, Ning Gao, Xinru Wang, Jian Peng, Hongkui Wei

**Affiliations:** ^1^ Department of Animal Nutrition and Feed Science College of Animal Science and Technology Huazhong Agricultural University Wuhan 430070 China; ^2^ The Cooperative Innovation Center for Sustainable Pig Production Wuhan 430070 China; ^3^ Frontiers Science Center for Animal Breeding and Sustainable Production Wuhan 430070 China; ^4^ Hubei Hongshan Laboratory Wuhan 430070 China

**Keywords:** bile acid, early life, gamma‐delta T, microbiome, N6‐methyl‐adenosine, retinoic acid‐related orphan receptor, type 3 innate lymphoid cell

## Abstract

This study investigates how microbiome colonization influences the development of intestinal type 3 immunity in neonates. The results showed that reduced oxygen levels in the small intestine of neonatal rats induced by *Saccharomyces boulardii* accelerated microbiome colonization and type 3 immunity development, which protected against *Salmonella enterica* serovar Typhimurium infection. Microbiome maturation increased the abundance of microbiome‐encoded bile salt hydrolase (*BSH*) genes and hyocholic acid (HCA) levels. Furthermore, reducing oxygen levels in the intestine increased the abundance of *Limosilactobacillus reuteri*, a bacterium encoding *BSH*, and promoted intestinal type 3 immunity. However, inhibition of BSH blocked the *L. reuteri‐*induced development of intestinal type 3 immunity. Mechanistically, HCA promoted the development of gamma‐delta T cells and type 3 innate lymphoid cells by stabilizing the mRNA expression of RAR‐related orphan receptor C via the farnesoid X receptor–WT1‐associated protein‐N6‐methyl‐adenosine axis. These results reveal that gut microbiota‐derived HCA plays a crucial role in promoting the development of intestinal type 3 immunity in neonates. This discovery introduces potential therapeutic avenues for strengthening intestinal immunity in early life or treating bacterial infections by targeting microbial metabolites.

## Graphical abstract

Reduction of intestinal oxygen levels in neonatal rats enhances *Lactobacillus* colonization, increasing bile salt hydrolase (BSH) activity and converting primary bile acids to hyocholic acid (HCA). HCA stabilizes *Rorc* mRNA via WTAP‐mediated m^6^A modification, promoting γδT cells and ILC3s development. This FXR‐modulated process enhances type 3 immunity and interleukin‐17A secretion, protecting against *Salmonella Typhimurium* infection.

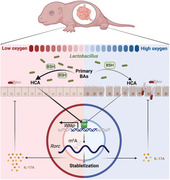

## 1 Introduction

Infections significantly contribute to neonatal mortality, which is largely attributed to the immature immune systems of neonates, which make them more vulnerable to infections than adults.^[^ ^1^, ^2^ ^]^ The neonatal period is a crucial stage for the development of the immune system. Numerous immune cells, including T cells^[^ ^3^ ^]^ and innate lymphoid cells (ILCs),^[^ ^4^ ^]^ are amplified in early life, playing a central role in fighting infections. Type 3 immunity is characterized by interleukin (IL)‐17A and IL‐22 expression, as well as the expression of the key transcription factor retinoic acid‐related orphan receptor (RORγt) by type 3 innate lymphoid cells (ILC3s), gamma‐delta T (γδT) cells, and T helper (Th) 17 cells.^[^ ^5^ ^]^ Type 3 immunity serves a vital role in defending against microbial infection and maintaining intestinal epithelial homeostasis.^[^ ^6^ ^–^ ^8^ ^]^

In mammals, the neonatal period is critical for microbiome colonization, which profoundly impacts the development of intestinal immune function. Early exposure to antibiotics leads to an immature microbiome and a weak type 3 immune system.^[^ ^9^, ^10^ ^]^ Moreover, germ‐free (GF) models showed that the function of type 3 immunity depends on the intestinal microbiome.^[^ ^3^, ^11^, ^12^ ^]^ Short‐chain fatty acid (SCFA) and tryptophan metabolism mediated by the microbiome supports ILC3 and T cell expansion and cytokine secretion.^[^ ^13^, ^14^ ^]^ The intestinal microbiome also influences host epigenetic modification.^[^ ^15^ ^]^ Microbial transfer to neonatal mice reverses the antibiotic‐induced reduction in histone modifications in the ileum.^[^ ^16^ ^]^ In addition, studies performed in antibiotic‐treated and GF mouse models have demonstrated that the levels of N6‐methyl‐adenosine (m^6^A) modification increase in the intestine when the microbiota is reduced or absent, suggesting that microbes modulate the m^6^A methylation of host mRNAs.^[^ ^17^, ^18^ ^]^ Mechanistically, the microbial metabolite folate regulates m^6^A modification levels by providing S‐adenosylmethionine, and the microbial metabolite butyrate participates in the tricarboxylic acid cycle, which leads to RNA demethylation.^[^ ^19^ ^]^ m^6^A is a major post‐transcriptional regulator of immune cells.^[^ ^20^ ^]^ However, whether the microbiome helps regulate m^6^A modification in intestinal immune cells in early life remains unknown.

The colonization of the gut microbiota in early life is regulated by factors such as delivery mode, environmental exposure, diet, and antibiotic use.^[^ ^21^, ^22^ ^]^ Oxygen is consumed by pioneer facultative anaerobic bacteria in the aerobic gut in early life, providing a niche for colonization by obligate anaerobes.^[^ ^23^ ^]^ However, the precise mechanisms through which microbiome colonization regulates type 3 immunity development and the post‐transcriptional modifications involved in this process remain poorly understood.

Here, we demonstrate that reducing oxygen levels in the rat intestine in early life facilitates colonization by the microbiome, which improves ability to combat *Salmonella enterica* serovar Typhimurium (*S*. Typhimurium) infection via a type 3 immune response. Hyocholic acid (HCA) enhanced RAR‐related orphan receptor C (*Rorc*) mRNA stability via WT1‐associated protein (WTAP)‐mediated N6‐methyl‐adenosine (m^6^A) by inhibiting farnesoid X receptor (FXR), and treatment with the FXR inhibitor GW4064 disrupted the HCA‐mediated promotion of type 3 immunity development. *Limosilactobacillus reuteri* increased HCA levels and enhanced type 3 immune response in a bile salt hydrolase (BSH)‐dependent manner. The findings of this study reveal novel mechanisms by which early‐life microbiota shape type 3 immunity, with broader implications for understanding neonatal immune maturation and developing therapeutic interventions to strengthen intestinal immunity or combat bacterial infections in early life.

## 2 Results

### 2.1 Oral Administration of *S. boulardii* Reduces Oxygen Levels in the Intestinal Lumen and Facilitates Maturation of the Intestinal Microbiome in Neonatal Rats

**Figure 1:**
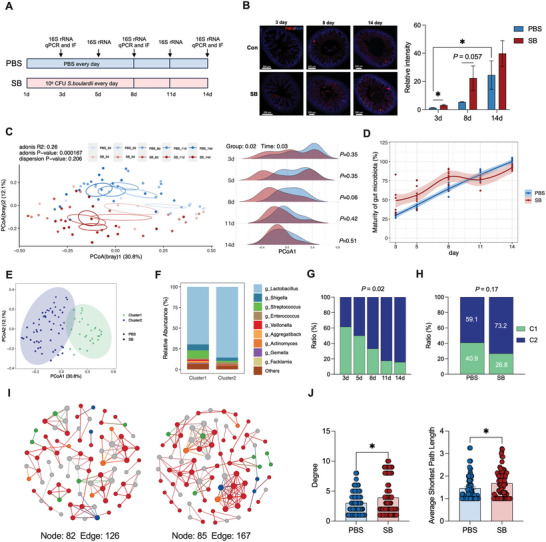
Early administration of *S. boulardii* reduces oxygen levels in the intestinal lumen and facilitates maturation of the intestinal microbiome. A) *S. boulardii* treatment experimental timeline. B) Representative images of immunofluorescent staining for hypoxyprobe‐1 (red) and nuclei (blue) in ileal tissue, and quantification of staining intensities, *n* = 3–4 (scale bars: 200 µm). C) PCoA plot of rats after PBS and *S. boulardii* treatment at days 3, 5, 8, 11, and 14 after birth based on Bray–Curtis analysis. The density plot shows a comparison of the PcoA1 of the PBS and SB groups at days 3, 5, 8, 11, and 14 after birth, *n* = 6–10. D) Intestinal microbiome maturity curve of rats after PBS and *S. boulardii* treatment, as assessed by a random forest model, *n* = 6–10. E) Two clusters were identified by an unsupervised clustering algorithm. Cluster 2 was identified as a mature type, and Cluster 1 was identified as an immature type, *n* = 86. F) Intestinal microbiome composition of Cluster 2 and Cluster 1 at the genus level. G,H) Proportions of the two clusters at the five sampling times, *n* = 13–19 (G) and comparison of the proportions of the two clusters in rats after PBS and *S. boulardii* treatment, *n* = 41 and 44 (H). The significance of dissimilarity was calculated by chi‐squared test. I) Network analysis of the intestinal microbiome of rats after PBS and *S. boulardii* treatment, *n* = 41 and 44. J) Comparison of network degree and average shortest path length. Data represent mean ± SEM. *p < 0.05. The difference between rats after PBS and *S. boulardii* treatment was analyzed by two‐tailed unpaired Student's *t*‐test, except for (D), (E), (G), and (H).

To investigate the effects of reducing intestinal oxygen levels on microbial colonization in early life, we administered the facultative anaerobic microorganism *S. boulardii* to neonatal rats and assessed changes in the gut oxygen status and gut microbial colonization (**Figure** **1**A). Results showed that intestinal oxygen levels gradually declined after *S. boulardii* administration. Early administration of *S. boulardii* significantly reduced oxygen levels at 3 days and induced a decreasing trend in oxygen levels at 8 days (Figure 1B). Hypoxic environments strongly stabilize HIF‐1α and transactivate HIF target genes such as *Bnip3l* in epithelial cells.^[^ ^24^ ^]^ In the current study, early administration of *S. boulardii* increased the mRNA expression levels of HIF‐1α and its target genes, including *Pfkb3*, *Defb1*, *Muc3*, *Bnip3l*, *Slc2a1*, and *Tff3*, in the ileum at 3, 8, and 14 days, indicating that the intestinal lumen was a relatively hypoxic environment (Figure S2A–C, Supporting Information). Early administration of *S. boulardii* significantly increased the alpha‐diversity index Chao1 of the microbiome at 3 days after birth and significantly decreased this index at 8 days after birth (Figure S3A, Supporting Information). Meanwhile, significant differences in the beta‐diversity of the microbiome were found between the *S. boulardii* (SB) and phosphate‐buffered saline (PBS) groups, especially after 5 days (Figure S3B–G, Supporting Information).

To evaluate the effect of the *S. boulardii* on microbiome maturation, we performed permutational multivariate analysis of variance on Bray–Curtis dissimilarities among the samples. Both animal age and *S. boulardii* administration significantly affected the microbiome composition (Figure 1C). Further, a transition in the microbiome was observed over time on the PCoA1 axis, which was accelerated in the neonates that received *S. boulardii*, particularly at 8 days (Figure 1C). Furthermore, a random forest analysis was conducted to generate a best‐fit curve for microbiome maturity, which showed that *S. boulardii* administration accelerated microbiome development (Figure 1D). Subsequently, an unsupervised clustering algorithm was used, which classified the neonatal intestine microbiome into two clusters that corresponded to the maturation trajectory observed in the PCoA plot (Figure 1E). Considering that the microbiome clusters gradually shifted toward the left side of the PCoA1 axis as they aged (Figure 1C), Cluster 2 (left) was deemed as the mature type and Cluster 1 (right), the immature type. At the genus level, *g_Lactobacillus* was enriched in Cluster 2 (mature type), whereas *g_Streptococcus* was enriched in Cluster 1 (immature type) (Figure 1F). The ratio of the mature microbiome (Cluster 2) to the immature microbiome (Cluster 1) gradually increased with age (Figure 1G), and a higher proportion of mature microbiome (Cluster 2) components was observed in the SB group compared with that in the PBS group (Figure 1H). Early‐life microbial maturation often involves an increase in community diversity and interaction network complexity.^[^ ^25^ ^]^ To further assess the parameters of the microbial community ecology, we performed a co‐occurrence network analysis to compare complexity and stability between the PBS and SB groups. The SB group had more nodes and edges than the PBS group (Figure 1I). At the node level, the degree and average shortest path length of the SB group were significantly higher than those of the PBS group (Figure 1J). Collectively, these findings suggest that *S. boulardii* accelerates the assembly of a more mature and stable microbiome.

### 2.2 Oral Administration of *S. boulardii* Facilitates the Maturation of Type 3 Immune Cells

**Figure 2:**
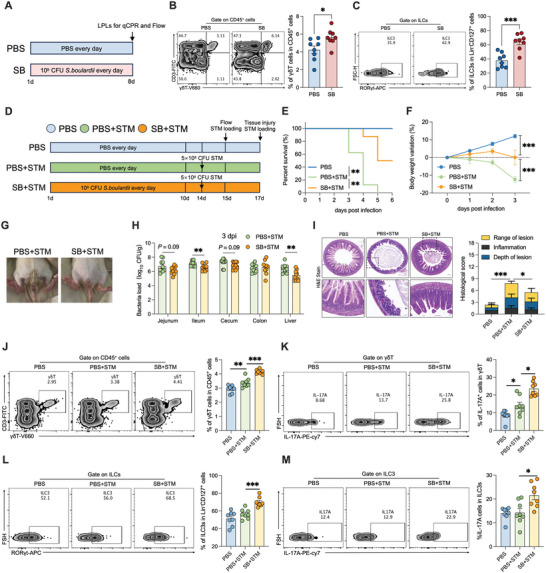
Oral administration of *S. boulardii* facilitates maturation of type 3 immune cells and enhances resistance to *S*. Typhimurium infection. A) *S. boulardii* treatment experimental timeline. B,C) Representative FACS plots and percentages of γδT cells and ILC3s in small intestine LPLs from rats, *n* = 8. D) *S. boulardii* treatment and *S*. Typhimurium infection experimental timeline. E,F) Survival curves and body weight changes in the PBS, PBS+STM, and SB+STM groups, *n* = 6. G) Representative diarrhea images. H) STM burden in the jejunum, ileum, cecum, colon, and liver at 3 dpi, *n* = 10–12. I) Representative H&E images showing histological scores, *n* = 5–6. J,K) Representative FACS plots and percentages of γδT and IL‐17A^+^γδT cells in small intestine LPLs from rats, *n* = 8. L,M) Representative FACS plots and percentages of ILC3s and IL‐17A^+^ILC3 cells in small intestine LPLs from rats, *n* = 8. Data represent mean ± SEM. *p < 0.05; **p < 0.01; ***p < 0.001. Differences between two groups were analyzed by two‐tailed unpaired Student's *t*‐test, except for (E) and (F).

Early establishment of the intestinal microbiome is crucial for immune cell maturation. Therefore, we detected the transcriptional levels of three transcription factors *Tbx21*, *Gata3*, and *Rorc*, which direct the maturation of type 1, 2, and 3 immune cells, respectively,^[^ ^26^ ^]^ in intestinal lamina propria lymphocytes (LPLs) (**Figure** **2**A). Only *Rorc* mRNA levels were elevated after early administration of *S. boulardii* (Figure S4A, Supporting Information). Subsequently, we examined the proportions of RORγt‐expressing type 3 immune cells, Th17 cells, γδT cells, and ILC3s among LPLs. The proportions of γδT cells and ILC3s (Figure 2B,C), but not Th17 cells (Figure S4B, Supporting Information), were significantly elevated after early administration of *S. boulardii*. Secretion of the type 3 immune cytokines IL‐17A and IL‐22 by γδT cells and ILC3s, respectively, also increased after early administration of *S. boulardii* (Figure S4C–F, Supporting Information). The proportions of T cells, B cells, macrophages, CD4^+^T cells, and CD8^+^T cells did not change (Figure S4G,H, Supporting Information). Taken together, these findings suggest that early administration of *S. boulardii* selectively promotes the maturation of type 3 immune cells, particularly γδT cells and ILC3s.

### 2.3 Oral Administration of *S. boulardii* Confers Protection Against *S*. Typhimurium Infection

We investigated whether oral administration of *S. boulardii* protects against *Salmonella* infection (Figure 2D). All control rats died from *S*. Typhimurium infection within 5 days post‐infection (dpi), whereas approximately 50% of the rats that received *S. boulardii* survived (Figure 2E). Early administration of *S. boulardii* reduced weight loss and diarrhea in the neonatal rats after *S*. Typhimurium infection (Figure 2F,G). *S*. Typhimurium colonization of the small intestine (jejunum and ileum) but not of the large intestine (cecum and colon) or liver was reduced at 1 dpi by *S. boulardii* administration (Figure S5A, Supporting Information). However, *S. boulardii* administration significantly reduced *S*. Typhimurium colonization of the ileum and liver at 3 dpi (Figure 2H). In addition, *S*. Typhimurium infection induced extensive epithelial damage and inflammatory cell infiltration, which were alleviated by *S. boulardii* administration, in the neonatal rats (Figure 2I).

A previous study demonstrated that *S. boulardii* directly restricts *S*. Typhimurium expansion in the intestine.^[^ ^27^ ^]^ To confirm this, we measured *S. boulardii* in the ileal contents and found that it was no longer detectable 4 days after the last administration (Figure S5B, Supporting Information). This result suggests that *S. boulardii* did not directly compete with *S*. Typhimurium for colonization of the intestine. Subsequently, we examined the type 3 immune response in neonatal rats after *S*. Typhimurium infection. *Rorc* and *Il17a* mRNA levels were upregulated in the SB group, but no significant difference in *Il22* mRNA levels was found between the SB and PBS groups (Figure S5C, Supporting Information). Similarly, the levels of IL‐17A but not IL‐22 were upregulated in the SB group compared with the PBS group (Figure S5D, Supporting Information). Moreover, *Il17a* mRNA levels correlated with the *S*. Typhimurium load in the ileum (Figure S5E, Supporting Information). Additionally, the type 3 immune response, including the percentages of γδ T cells and ILC3s and their ability to secrete IL‐17A, was enhanced by *S. boulardii* administration (Figure 2J–M). These results demonstrate that *S. boulardii* reduces *S*. Typhimurium load, alleviates weight loss and diarrhea, and increases the proportions of IL‐17A‐secreting γδT cells and ILC3s.

### 2.4 Transplantation of the Whole Intestinal Microbiota from the *S. boulardii*‐Administered Rats to Neonatal Rats Accelerates Gut Microbial Maturation and Enhances Resistance to *S*. Typhimurium Infection

**Figure 3:**
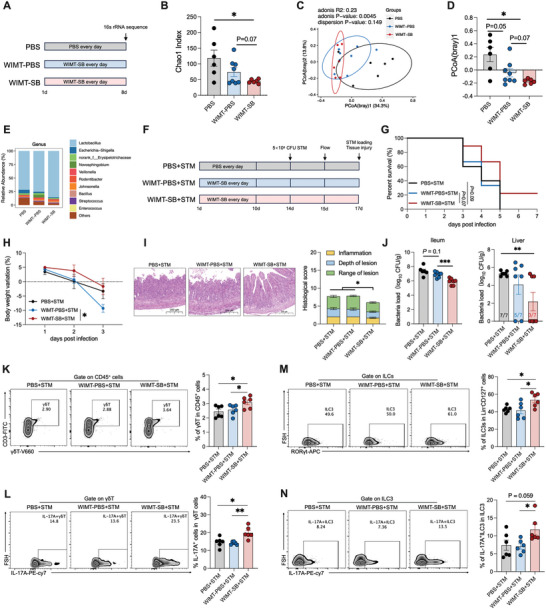
Microbial transfer from the SB group accelerates microbial maturation and enhances resistance to *S*. Typhimurium infection. A) WIMT treatment experimental timeline. B) α‐diversity (as assessed by Chao1 index) of the ileal microbiome, *n* = 6–8. C,D) PCoA plot of rats from three groups based on Bray–Curtis analysis. The significance of dissimilarity was calculated by adonis and dispersion analyses, *n* = 6–8. E) Relative abundance of genera. F) WIMT treatment and *STm* infection experimental timeline. G,H) Survival curves and bodyweight changes in the PBS+STM, WIMT‐PBS+STM, and WIMT‐SB+STM groups, *n* = 9. I) Representative H&E images showing histological scores, *n* = 7. J) STM burden in the ileum and liver, *n* = 7. K,L) Representative FACS plots and percentages of γδT and IL‐17A^+^γδT cells in small intestine LPLs from rats, *n* = 8. M,N) Representative FACS plots and percentages of ILC3s and IL‐17A^+^ILC3s in small intestine LPLs from rats, *n* = 8. Data represent mean ± SEM. *p <  0.05; **p < 0.01; ***p < 0.001. Differences between the two groups were analyzed by two‐tailed unpaired Student's *t*‐test, except for (C), (G), and (H).

To investigate whether the development of intestinal type 3 immunity depends on the microbiome, we conducted whole intestinal microbial transplantation (WIMT) (**Figure** **3**A), which is conducive to donor small intestinal microbiome colonization than fecal microbiota transplantation (FMT).^[^ ^28^ ^]^ Briefly, we orally administered microbiota from the jejunum, ileum, cecum, and colon to rats on days 1 to 8. The Chao 1 index in the WIMT‐SB group was lower than that in the PBS group (Figure 3B) but was similar to that in the WIMT‐PBS group. β‐diversity analysis showed that the microbial composition was different among the three groups (Figure 3C,D). At the genus level, the relative abundance of *Lactobacillus* increased after WIMT‐SB treatment (Figure 3E).

We explored the effect of WIMT on susceptibility to *S*. Typhimurium infection (Figure 3F). All neonatal rats from the PBS and WIMT‐PBS groups died of *S*. Typhimurium infection within 5 days post‐infection, whereas approximately 20% of the neonatal rats from the WIMT‐SB group survived (Figure 3G). Compared with WIMT‐PBS, WIMT‐SB significantly alleviated body weight loss and decreased *S*. Typhimurium load in the neonatal rats post‐infection (Figure 3G–I). Additionally, the rats in the WIMT‐SB+*S*. Typhimurium (STM) group exhibited lower histological scores than those in the PBS+STM and WIMT‐PBS+STM groups (Figure 3J). Furthermore, the percentages of γδT cells and ILC3s, as well as their ability to secrete IL‐17A, was enhanced by WIMT‐SB treatment (Figure 3K–N). Overall, these findings show that WIMT from the SB group accelerates microbial maturation and enhances resistance to *S*. Typhimurium infection in neonatal rats, indicating that the development of intestinal type 3 immunity depends on the microbiome.

### 2.5 Maturation of the Gut Microbiome Facilitates Type 3 Cell Function through Bile Acid Metabolism

**Figure 4:**
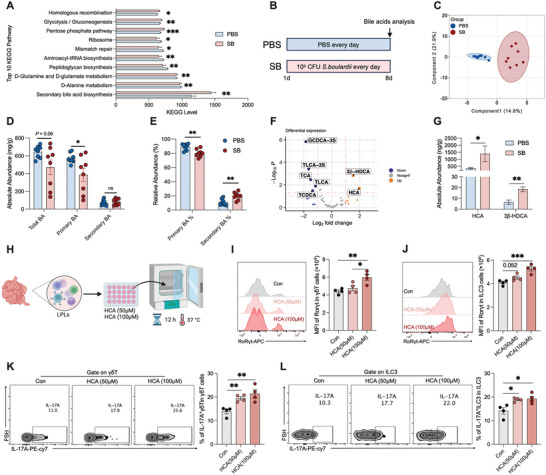
Gut microbiome maturation facilitates type 3 cell function through BAs metabolism. A) Top 10 predicted microbial functions based on PICRUSt2 after PBS and *S. boulardii* treatment, *n* = 44 and 41, respectively. B) *S. boulardii* treatment and BA analysis experimental timeline. C) OPLS‐DA score plot of the ileal content BA profiles in rats after PBS or *S. boulardii* treatment at 8 days, *n* = 8. D) Absolute abundance of primary, secondary, and total BAs, *n* = 8. E) Relative abundance of secondary and primary BAs, *n* = 8. F) Volcano plot analysis of different BAs, with a threshold of FC > 2 or FC < 0.5, and *P* < 0.05, *n* = 8. G) Absolute abundance of HCA and 3β‐HDCA, *n* = 8. H) Workflow of in vitro LPL treatment with HCA. I,J) Representative FACS plots and MFI of RORγt expression in γδT cells and ILC3s from small intestine LPLs from rats after HCA treatment in vitro, *n* = 4. K,L) Representative FACS plots and percentages of IL‐17A^+^γδT cells and IL‐17A^+^ILC3s in small intestine LPLs from rats after HCA treatment in vitro, *n* = 4. Data represent mean ± SEM. *p < 0.05; **p < 0.01; ***p < 0.001. Differences between the two groups were analyzed by two‐tailed unpaired Student's *t*‐test, except for (C) and (F).

To explore the potential functional capabilities of the mature microbiome, we performed Kyoto Encyclopedia of Genes and Genomes (KEGG) functional prediction using phylogenetic investigation of communities by reconstruction of unobserved states (PICRUSt2) analysis. We found that the biosynthesis of secondary bile acids (BAs) was the most enriched pathway and had the highest fold change among the top 10 KEGG pathways (**Figure** **4**A). Therefore, we assessed the BA profiles in the ileum of the rats in the PBS and SB groups by LC‐MS/MS (Figure 4B). A total of 63 BAs were detected, with a clear separation between the BA profiles of the SB and PBS groups, by orthogonal partial least squares discriminant analysis (OPLS‐DA) (Figure 4C). Overall, the absolute levels of total and primary BAs decreased (Figure 4D), whereas the relative levels of primary BAs decreased significantly, and the relative levels of secondary BAs increased significantly, in the SB group compared with the PBS group (Figure 4E). Among the 63 BAs measured, seven exhibited significantly different levels between the two groups (fold change >2, *p* < 0.05). Specifically, 3β‐hyodeoxycholic acid (HDCA) and HCA levels increased, whereas glycochenodeoxycholic acid‐3S, taurolithocholic acid‐3S, taurocholic acid (TCA), lithocholic acid, and taurochenodeoxycholic acid levels decreased in the SB group compared with the PBS group (Figure 4F). The absolute HCA level was significantly higher than the absolute 3β‐HDCA level (Figure 4G). Moreover, 3β‐HDCA was not detected in samples from two of the rats that were analyzed. This result suggests that the increase in 3β‐HDCA levels may not be universally present in all rats and therefore not be generally significant. Thus, we hypothesized that HCA is the main factor contributing to the crosstalk between the microbiome and host immune system maturation. To verify this, we isolated LPLs from the small intestine of rats and incubated them ex vivo with HCA (Figure 4H). HCA treatment increased the mean fluorescence intensity of RORγt in γδT cells and ILC3s (Figure 4I,J). It also increased IL‐17A secretion by ILC3s and γδT cells (Figure 4K,L). Taken together, these findings suggest that secondary BA biosynthesis increases in the intestinal microbiome after *S. boulardii* administration and that HCA enhances the function of type 3 immune γδT cells and ILC3s.

### 2.6 *Lactobacillus reuteri* Modulates BA Metabolism and Elevates HCA Levels in Neonatal Rats

**Figure 5:**
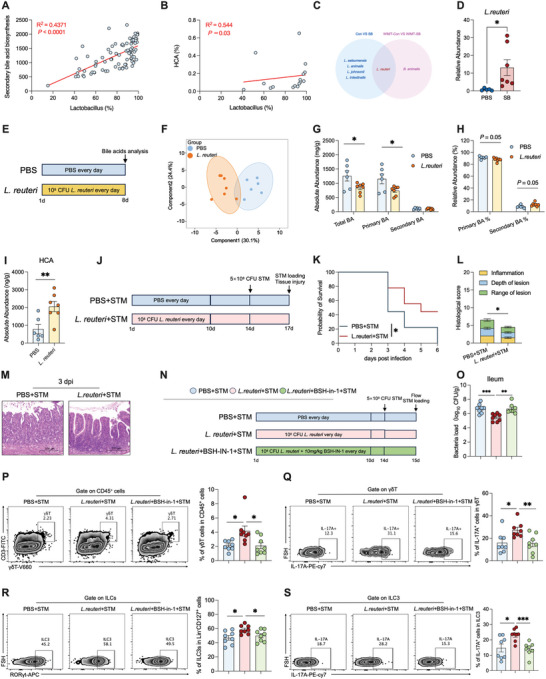
*L. reuteri* enhances the type 3 immune response to *S*. Typhimurium infection. A) Correlation between the relative abundance of *Lactobacillus* and secondary BA biosynthesis in the rat ileum, *n* = 85. B) Correlation between the relative abundance of *Lactobacillus* and the relative abundance of HCA in the rat ileum, *n* = 16. C) Venn diagram of the enriched species in the two experiments. D) RT‐qPCR analysis of the relative abundance of *L. reuteri* in the ileum of PBS and SB rats, *n* = 5–7. E) *L. reuteri* treatment for BA analysis experimental timeline. F) OPLS‐DA score plot of the ileal BA profiles between the PBS and *L. reuteri* groups at 8 days, *n* = 6–7. G) Absolute abundance of primary, secondary, and total BAs in the ileal contents, *n* = 6–7. H) Relative abundance of secondary and primary BAs in the ileal contents, *n* = 6–7. I) Absolute abundance of HCA, *n* = 6–7. J) *L. reuteri* treatment and *STm* infection experimental timeline. K) Survival curves of the *STm* and *L. reuteri* + STM groups. The significance of dissimilarity was calculated by log‐rank (Mantel–Cox) test, *n* = 9. L,M) Representative H&E images showing histological scores in the *STm* and *L. reuteri* + *STm* groups. The significance of dissimilarity was calculated by *t*‐test, *n* = 6. N) *L. reuteri* and BSH inhibitor treatment experimental timeline. O) STM burden in the ileum of the PBS + STM, *L. reuteri* + STM, and *L. reuteri* + BSH‐in + STM groups at 1 dpi, *n* = 8. P,Q) Representative FACS plots and percentages of γδT and IL‐17A^+^γδT cells in small intestine LPLs, *n* = 8. R,S) Representative FACS plots and percentages of ILC3s and IL‐17A^+^ILC3s in small intestine LPLs, *n* = 8. Data represent mean ± SEM. *p < 0.05; **p < 0.01. Differences between the two groups were analyzed by two‐tailed unpaired Student's *t*‐test, except for (A), (B), (F), and (K).

Next, we aimed to identify the bacteria contributing to the increased levels of HCA in neonatal rats. Our analysis revealed a positive correlation between *Lactobacillus* abundance and secondary BA biosynthesis (**Figure** **5**A). Moreover, *Lactobacillus* abundance correlated positively with HCA levels (Figure 5B). The first step in the metabolism of secondary BAs is mediated by BSH. Considering that *Lactobacillus* expresses *BSH*, we compared the relative abundance of *BSH* in the microbiome between the PBS and SB groups. Compared with the PBS group, the SB group showed a tendency toward increased *BSH* relative abundance (Figure S6A, Supporting Information). All enriched amplicon sequence variant (ASV) sequences in the SB group were annotated as *Lactobacillus* (Figure S6B, Supporting Information). Additionally, *L. reuteri* and *B. animalis* were enriched in the WIMT‐SB group (Figure S6C, Supporting Information), whereas only *L. reuteri* was enriched in the SB and WIMT‐SB groups (Figure 5C). *L. reuteri* enrichment in the SB group at 8 days was validated by quantitative real‐time polymerase chain reaction (qPCR) (Figure 5D).

To test the effect of *L. reuteri* on BA metabolism, we orally administered *L. reuteri* or PBS to neonatal rats and analyzed the BA profiles in their intestines 8 days after birth (Figure 5E). OPLS‐DA analysis demonstrated a clear separation in the BA profiles between the two groups (Figure 5F). The absolute levels of total and primary BAs decreased (Figure 5G). Furthermore, the relative levels of primary BAs showed a decreasing trend, whereas those of secondary BAs showed an increasing trend, after *L. reuteri* treatment (Figure 5H). Among the secondary BAs tested, HCA levels increased after *L. reuteri* treatment (Figure 5I; Figure S6D, Supporting Information). Collectively, our findings suggest that *L. reuteri* modulates BA profiles and increases HCA levels in the intestines of neonatal rats.

### 2.7 *Lactobacillus reuteri* Enhances the Type 3 Immune Response to *S*. Typhimurium Infection in a BSH‐Dependent Manner

To verify the protective effect of *L. reuteri* against *S*. Typhimurium, we evaluated rat phenotypes after *S*. Typhimurium infection (Figure 5J). *L. reuteri* administration mitigated body weight loss (Figure S7A, Supporting Information), mortality (Figure 5K), and diarrhea (Figure S7B, Supporting Information) after *S*. Typhimurium infection. Additionally, *L. reuteri* administration reduced *S*. Typhimurium burden in the ileum and liver (Figure S7C, Supporting Information), as well as ileum damage (Figure 5L,M), at 3 dpi.

Subsequently, we treated rats that had received *L. reuteri* with the BSH inhibitor (BSH‐IN‐1) (Figure 5N). As expected, the *S*. Typhimurium burden in the ileum was restored to the level observed in the PBS+STM group after BSH‐IN‐1 treatment (Figure 5O). Moreover, the type 3 immune response, including the percentages of γδT cells and ILC3s and their secretion of IL‐17A, was abolished in the *L. reuteri*+BSH‐IN‐1+STM group (Figure 5P–S). Collectively, these results demonstrate that *L. reuteri* protects against *S*. Typhimurium infection by promoting a type 3 immune response in a BSH‐dependent manner.

Next, we want to evaluate of bile acids metabolism in *S. boulardii* administration‐induced facilitation of type 3 immunity (Figure S8A, Supporting Information). Although SB+BSH‐IN+STM did not significantly affect body weight variation, it increased the S. Typhimurium burden and suppressed the IL‐17A response compared to SB+STM. Furthermore, we observed a trend indicating differences between the SB+BSH‐IN+STM and PBS+STM groups in the *S*. Typhimurium burden and IL‐17A response (Figure S8B–D, Supporting Information). Collectively, these results demonstrate that BSH inhibition was found to partially suppress the *S. boulardii* ‐induced type 3 immune response and its protective effects against *S*. Typhimurium infection.

### 2.8 HCA Enhances the Function of Type 3 Immune Cells by Inhibiting FXR

**Figure 6:**
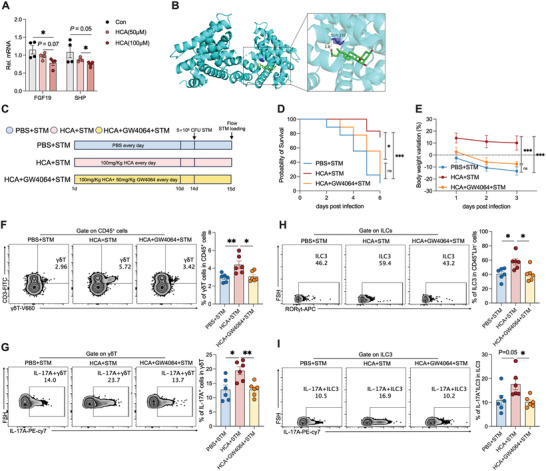
HCA enhances the function of type 3 immune cells via inhibition of FXR. A) RT‐qPCR analysis of mRNAs encoded by the FXR target genes *Fgf19* and *Shp* in LPLs after HCA treatment in vitro, *n* = 4. B) Molecular docking analysis shows that HCA binds to FXR SER‐332. C) HCA and GW4064 treatment and STM infection experimental timeline. D,E) Survival curves and bodyweight changes in the PBS+STM, HCA+STM, and HCA+GW4064+STM groups, *n* = 9–12. F,G) Representative FACS plots and percentages of γδT and IL‐17A^+^γδT cells in small intestine LPLs, *n* = 6. H,I) Representative FACS plots and percentages of ILC3s and IL‐17A^+^ILC3s in small intestine LPLs, *n* = 6. Data represent mean ± SEM. *p < 0.05; **p < 0.01; ***p < 0.001; ns, no significance. Differences between the two groups were analyzed by two‐tailed unpaired Student's *t*‐test, except for (E) and (F).

HCA is an antagonist of FXR,^[^ ^29^ ^]^ and blocking FXR activation promotes IL‐17A production by ILC3s.^[^ ^30^ ^]^ Therefore, we hypothesized that HCA enhances the function of type 3 immune cells by inhibiting FXR. Results showed that treating LPLs with HCA decreased the expression of FXR target genes (*Fgf19* and *Shp*) (**Figure** **6**A). To fully elucidate the interaction between HCA and FXR, we conducted a molecular docking study. Docking results showed that HCA could bind well to FXR through visible hydrogen bonds and strong electrostatic interactions, with a low binding energy of ‐7.991 kcal/mol. The hydrogen bonding between the residue SER‐332 and HCA may play an important role in the observed interaction (Figure 6B).

Subsequently, we orally administered HCA and the FXR agonist GW4064^[^ ^31^ ^]^ to neonatal rats (Figure 6C). HCA alleviated the diarrhea, weight loss, and mortality induced by *S*. Typhimurium infection, whereas simultaneous administration of GW4064 with HCA reduced this effect (Figure 6D,E; Figure S9A,B, Supporting Information). Further, the type 3 immune response, including the increase in γδT cells and ILC3s percentages and their secretion of IL‐17A, was enhanced by HCA and blocked by GW4064 treatment (Figure 6F–I). To further demonstrate that HCA plays a protective role against *S*. Typhimurium infection via type 3 immunity, we orally administered HCA and the RORγt inhibitor GSK805 to neonatal rats (Figure S9C, Supporting Information). The results showed that GSK805 treatment significantly weakened the ability of HCA to mitigate *S*. Typhimurium invasion of the ileum (Figure S9D, Supporting Information) and reduced the ability of HCA to induce a type 3 immune response (Figure S9E, Supporting Information). Collectively, these findings suggest that HCA protects against *S*. Typhimurium infection by promoting a type 3 immune response via inhibition of FXR.

### 2.9 HCA Enhances *Rorc* mRNA Stability by Inhibiting FXR‐Induced *Wtap* Transcription

*Rorc* is a vital factor in the development of type 3 immunity, and *S. boulardii* promotes *Rorc* expression in LPLs. Thus, we investigated the effect of HCA or glycine‐conjugated β‐muricholic acid (Gly‐MCA) treatment on *Rorc* expression in LPLs. *Rorc* expression increased in the LPLs from the rats treated with HCA or the FXR inhibitor Gly‐MCA (Figure S10A, Supporting Information). Although FXR is a nuclear receptor, previous studies have shown that FXR does not transcriptionally regulate *Rorc* in HEK293T cells.^[^ ^30^ ^]^ Therefore, we posited that other epigenetic mechanisms contribute to the FXR‐mediated regulation of *Rorc* expression. m^6^A is one of the most common modifications of RNA molecules in mammalian cells and broadly affects immune responses.^[^ ^32^, ^33^ ^]^ Furthermore, m^6^A modification in the intestine can be regulated by the microbiome.^[^ ^17^, ^18^ ^]^

To verify the importance of m^6^A modification in microbiome development and immune cells, we first detected changes in m^6^A modification in LPLs. Global m^6^A levels in LPL mRNAs were lower in the 14‐day‐old rats than in the 3‐day‐old rats (Figure S10B, Supporting Information), suggesting that m^6^A modification plays a vital role in the development of immune cells. Moreover, *S. boulardii* or *L. reuteri* administration decreased m^6^A levels in LPLs (Figure S10C,D, Supporting Information). We hypothesized that FXR affects m^6^A modification through transcriptional regulation. Therefore, we detected the mRNA levels of m^6^A‐related enzymes and found that the expression levels of three m^6^A reader–encoding genes, *Mettl3*, *Mettl14*, and *Wtap*, were downregulated after *S. boulardii* administration (Figure S10E, Supporting Information). In addition, treatment with HCA and the FXR inhibitor Gly‐MCA decreased the levels of m^6^A and *Wtap* but not *Mettl3* and *Mettl14* in LPLs (Figure S10F, Supporting Information). Moreover, *L. reuteri* administration decreased *Wtap* levels in LPLs (Figure S10G, Supporting Information).

**Figure 7:**
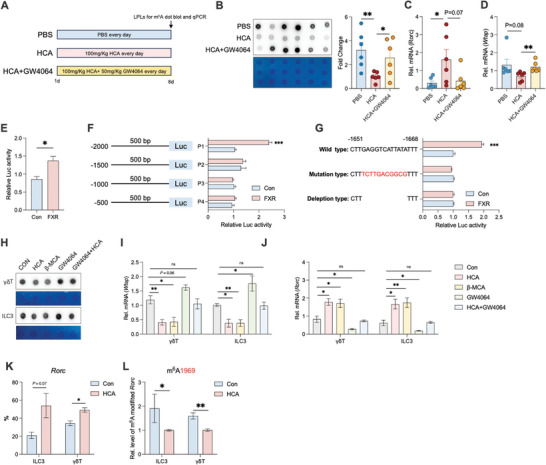
HCA enhances *RORC* mRNA stability. A) HCA and GW4064 treatment experimental timeline. B) Dot blot to detect the m^6^A levels of mRNA isolated from the total RNA of LPLs isolated from rats in the PBS, HCA, and HCA+GW4064 groups, *n* = 6. C,D) RT‐qPCR analysis of *Wtap* and *Rorc* mRNA levels in LPLs isolated from rats, *n* = 6. E) *Wtap* promoter reporter was co‐transfected with CMV‐FXR or empty vector (pReceiver‐M35) into 293T cells, and the promoter activity was determined as the ratio of firefly/Renilla luciferase activities. The dual‐luciferase activity was measured after 24 h of transfection, *n* = 3. F) Relative promoter activity of the *Wtap* promoter reporter (P1‐P4), *n* = 3. G) Relative promoter activity of the WT *Wtap* promoter and the mutant *Wtap* and *Wtap* deletion constructs, *n* = 3. H) Dot blot analysis of m^6^A levels of mRNA isolated from the total RNA of rat intestinal γδT cells and ILC3s with HCA, β‐MCA, GW4064 treatment. I,J) RT‐qPCR analysis of *Wtap* and *Rorc* mRNA levels in rat intestinal γδT cells and ILC3s with with HCA, β‐MCA, GW4064 treatment, *n* = 3. K) *Rorc* mRNA stability. RT‐qPCR analysis of *Rorc* mRNA levels in rat intestinal γδT cells and ILC3s with or without HCA treatment from 0 to 5 h, *n* = 3. L) m^6^A modification of site 1969 of the *Rorc* mRNA, as detected using an Epi‐SELECTTM m^6^A site identification kit, *n* = 3. Data represent mean ± SEM. *p < 0.05; **p < 0.01; ***p < 0.001. Differences between the two groups were analyzed by two‐tailed unpaired Student's *t*‐test.

To determine whether the change in m^6^A RNA levels depended on FXR, we treated rats with both HCA and the FXR agonist GW4064 (**Figure** **7**A). The results showed that GW4064 attenuated the HCA‐induced decrease in m^6^A modification, decrease in *Wtap* expression, and increase in *Rorc* expression (Figure 7B–D). We also predicted specific binding of FXR to the *Wtap* promoter using the Jasper database. To confirm whether FXR transcriptionally regulates *Wtap*, we constructed a luciferase reporter plasmid containing the human *Wtap* promoter and an FXR cDNA plasmid to perform a dual‐luciferase assay. FXR overexpression increased luciferase expression from the *Wtap* reporter (Figure 7E). To identify the region responsible for FXR‐mediated transactivation, we created constructs containing fragments of the human *Wtap* promoter (P1‐P4) and co‐transfected each of them into HEK293T cells with an FXR overexpression plasmid. As shown in Figure 7F, FXR overexpression significantly enhanced the activity of the P1 promoter (−2000 bp to −1500 bp) but not the P2–P4 promoters. Mutating or deleting the P1 site abolished the FXR‐mediated transactivation of promoter activity (Figure 7G). These results suggest that FXR plays a crucial role in WTAP transactivation.

To determine whether the m^6^A pathway contributes to the HCA‐mediated regulation of ILC3s and γδT cells, we sorted ILC3s and γδT cells and treated them with HCA, β‐MCA, and GW4064. As expected, HCA and FXR inhibitor β‐MCA reduced global m^6^A levels and *Wtap* expression, and increased *Rorc* expression in ILC3s and γδT cells (Figure 7H–J). FXR agonist GW4064 increased global m^6^A levels and *Wtap* expression, and decreased *Rorc* expression in ILC3s and γδT cells (Figure 7H–J). This study investigates how microbiome colonization influences the development of intestinal type 3 immunity in neonates. Further, GW4064 reversed the function of HCA (Figure 7H–J). An RNA decay assay showed that HCA treatment slowed the decrease in *Rorc* transcription in ILC3s and γδT cells (Figure 7K). Twelve potential m^6^A sites in the *Rorc* mRNA sequence were predicted using SRAMP, and decreased m^6^A modification of *Rorc* in the HCA‐treated ILC3s and γδT cells was verified using Epi‐SELECTTM (Figure 7L). Collectively, these data indicate that FXR transcriptionally regulates the expression of the m^6^A methyltransferase WTAP, thereby affecting *Rorc* mRNA m^6^A modification and stability.

## 3 Discussion

The microbiome profoundly affects the immune system in early life, including elements such as ILCs, γδT cells, and Paneth cells.^[^ ^34^ ^]^ In the present study, we explored how colonization with the microbiome promotes the development of intestinal type 3 immunity in early life. We found that *L. reuteri*‐derived HCA plays a crucial role in promoting the development of intestinal type 3 immunity in the neonatal period. Our findings have important clinical significance for preventing or treating bacterial infections in newborns through microbial therapy.

Although studies in germ‐free models have demonstrated the vital role of the intestinal microbiome in type 3 immunity development,^[^ ^35^, ^36^ ^]^ research on the relationship between the microbiota and type 3 immunity has mainly been limited to adult animal models. For instance, segmented filamentous bacteria colonize the ileum of adult mice and promote Th17 cell expansion.^[^ ^37^ ^]^ A previous study reported that *Akkermansia muciniphila* promotes the development of RORγt‐positive innate and adaptive immune cell subsets in the early life of mice,^[^ ^38^ ^]^ although the underlying mechanism was not clearly delineated. Microbial metabolites broadly influence the function of type 3 immune cells in adult animals. For instance, SCFAs facilitate the expansion of Th17 cells and ILC3s in the intestine via free fatty acid receptor 2,^[^ ^13^ ^]^ and *Lactobacillus*‐derived indole serves as an AHR ligand to enhance IL‐22 transcription in ILC3s.^[^ ^14^ ^]^ BA metabolism, which involves the intestinal microbiome, also contributes to the functions of peripheral regulatory T cells,^[^ ^39^ ^]^ ILCs,^[^ ^40^ ^]^ and intestinal stem cells.^[^ ^41^ ^]^ In the present study, *Lactobacillus* contributed to HCA production and enhanced γδT cell and ILC3 development in early life by inhibiting FXR, which effectively prevented intestinal bacterial infections. FXR and TGR5 are receptors for various BAs;^[^ ^42^ ^]^ HCA inhibits FXR and activates TGR5.^[^ ^29^ ^]^ FXR deletion in ILCs induces ILC3s in the intestine to secrete IL‐17,^[^ ^30^ ^]^ and blocking TGR5 reduces IL‐22 secretion by ILC3s.^[^ ^43^ ^]^ In the current study, HCA enhanced *Rorc* transcription via FXR‐dependent m^6^A RNA methylation and promoted IL‐17A secretion by γδT cells and ILC3s, thereby protecting against *S*. Typhimurium infection. These results demonstrate that microbiome‐derived HCA promotes intestinal type 3 immunity in early life.

The gut microbiome affects the epigenetic modification of host molecules.^[^ ^19^ ^]^ Microbial‐derived butyrate regulates histone acetylation in tuft cells,^[^ ^44^ ^]^ B cells,^[^ ^45^ ^]^ T cells,^[^ ^46^ ^]^ and ILCs^[^ ^47^ ^]^ via histone deacetylase. In addition, studies performed in germ‐free mouse models showed that m^6^A modification in the intestine can be regulated by the microbiome.^[^ ^17^, ^18^ ^]^ Infection of tumor cells with *Fusobacterium nucleatum* increases METTL3 transcription, thereby affecting the level of m^6^A modification.^[^ ^48^ ^]^ However, research on how microbes affect m^6^A modification in the host intestine is still limited. Moreover, previous studies have shown that m^6^A demethylase ALKBH5 deficiency impairs ILC3 homeostasis, thereby increasing susceptibility to *Citrobacter rodentium* infection of the intestine.^[^ ^49^ ^]^ Our findings indicated that early‐life microbiome colonization decreased m^6^A levels in intestinal LPLs via HCA. High m^6^A levels disrupted the mRNA stability of the type 3 immune factor *Rorc* in early life, and HCA enhanced *Rorc* mRNA stability through m^6^A modification, thereby facilitating the amplification of type 3 immune cells in early life, highlighting interactions between the microbiome and immune cells.

The first step in the metabolism of secondary BAs is hydrolysis by BSH, and *Clostridium*, *Enterococcus*, *Bifidobacterium, Lactobacillus*, and *Bacteroidetes* all express BSH.^[^ ^50^, ^51^, ^52^ ^]^ The present results showed that *Lactobacillus* abundance was associated with secondary BA biosynthesis and that *Lactobacillus BSH* abundance was enriched in the SB group. A recent study has reported that *L. reuteri* and *L. plantarum* alter BA profiles and ameliorate gut microbiome dysbiosis in mice.^[^ ^53^ ^]^ In the present study, early‐life administration of *L. reuteri* altered BA profiles, especially enriching the secondary BA HCA. Further, the BSH antagonist BSH‐In‐1 weakened *L. reuteri* function. Previous studies have reported that *L. reuteri* regulates IL‐17 production by T cells and ILC3s via tryptophan and SCFA metabolism in the colons of adult mice.^[^ ^54^, ^56^ ^]^ Furthermore, *L. reuteri* induces ILC3s expansion via IgA in the neonatal small intestine.^[^ ^57^ ^]^ In the present study, secondary BA biosynthesis was involved in the connection between the microbiome and type 3 immunity in the small intestine. We speculate that this is related to physiological state, diet, and the intestinal segment.

The oxygen level in the intestinal lumen is a vital factor influencing colonization by the microbiome.^[^ ^58^, ^59^ ^]^ Colonocyte oxygenation induced by PPAR‐γ signaling inhibits *Enterobacteriaceae* growth, thereby ameliorating colitis.^[^ ^60^, ^61^ ^]^ Moreover, reducing oxygen levels protects against colonization by pathogenic bacteria.^[^ ^62^, ^63^ ^]^ In early life, gut oxygen levels gradually decrease with age,^[^ ^64^ ^]^ and the microbiome composition changes from aerobic to anaerobic bacteria.^[^ ^65^, ^66^, ^67^ ^]^ Our results confirmed this, showing that reducing oxygen levels promoted microbiome development in newborn rats and induced a shift from anaerobic to facultative anaerobic bacteria, dominated by *Lactobacillus*. Anaerobic bacteria such as *Bifidobacterium* cannot colonize the ileum because of the higher oxygen level in the small intestine than the large intestine.^[^ ^68^ ^]^ Consequently, our data underscore the significance of reducing intestinal oxygen levels in promoting microbiome colonization in early life. However, administration of *S. boulardii* promotes type 3 immunity and resistance to *S*. Typhimurium infection through mechanisms beyond microbial modulation. For instance, reduced oxygen levels can drive ILC3 expansion through HIF‐1α, enhancing resistance to *Clostridiodes difficile* infection.^[^ ^69^ ^]^

Three types of immune responses play an important role in defending against microbial infections.^[^ ^70^ ^]^ In the present study, type 3 response involving γδT cells and ILC3s was elevated in response to *S*. Typhimurium, whereas Th17 cell numbers did not change. We speculate that Th17 cells, which are adaptive immune cells, do not develop in neonates because of the lack of SFB,^[^ ^71^ ^]^ whereas γδT cells and ILC3s are innate immune cells that are abundant in neonatal mice.^[^ ^4^, ^8^ ^]^ IL‐17A is produced early after infection by pathogens, which indicates the presence of innate IL‐17A secretion pathways. For example, IL‐17A is expressed 5 h after inoculation of rhesus macaque ileal loops and 48 h after oral infection of mice with *Salmonella*.^[^ ^72^, ^73^ ^]^ In the present study, *Il17a* expression was elevated 24 h after *S*. Typhimurium infection, and *S. boulardii* promoted the secretion of IL‐17A but not IL‐22 in the small intestine. Further, *Il17a* levels in the intestine were inversely correlated with *S*. Typhimurium load. Collectively, these findings suggest that IL‐17A‐producing type 3 γδT cells and ILC3s play a crucial role in protecting against *S*. Typhimurium infection in early life.

This study had some limitations regarding mechanistic investigation and clinical translation. Although *Lactobacillus* contributed to HCA production in a BSH‐dependent manner, the entire metabolic pathway leading to HCA synthesis was not fully delineated. Other bacteria may interact synergistically with *Lactobacillus* to produce HCA. Further techniques such as bacterial co‐culture and multi‐omics are needed to investigate the underlying mechanisms. Furthermore, in addition to *Rorc*, other WTAP target genes may be involved in the regulation of ILC3s and T cells. meRIP‐seq studies conducted at the genome level may provide additional valuable evidence, and genetically engineered animals could also be helpful for verifying the mechanisms.

Taken together, our findings show that a hypoxic environment in the intestine promotes colonization by the microbiome, particularly *Lactobacillus* species, early in life. HCA produced by *Lactobacillus* suppresses FXR signaling and enhances *Rorc* stability through the FXR‐WTAP‐m^6^A axis, which leads to type 3 γδT cell and ILC3 expansion and increased resistance to *S*. Typhimurium infection of the intestine.

## 4 Experimental Section

### Animal Experiments

Animal welfare was monitored and experimental procedures were conducted in accordance with the Guide for the Care and Use of Laboratory Animals (Eighth Edition) and were approved by the ethical committee at Huazhong Agricultural University, ID Number: 202 401 050 001. Wistar rats were obtained from the Animal Experiment Center at Hubei Disease Prevention and Control Center (Wuhan, China). The rats were housed under specific pathogen‐free conditions in a temperature‐controlled room at 23 ± 2 °C and given free access to food and water.

For *S. boulardii* administration, pregnant Wistar rats were monitored daily until parturition. After birth, each litter was culled to 12 pups. On postnatal day 1 (PND1), the pups in each litter were randomly divided into two groups (PBS or SB). Rats in the PBS group were orally administered PBS once per day from PND1 to PND10; rats in the SB group were orally administered 1 × 10^9^ colony‐forming units (CFUs) of *S. boulardii* once per day from PND1 to PD10. The test product was Levucell SB (Lallemand SAS, Blagnac, France). On PND3, PND5, PND8, PND11, and PND14, two rats from each group from each litter were randomly chosen and sacrificed by CO_2_ inhalation.

For early‐life *S*. Typhimurium infection, the rats were infected on PND14 with 1 × 10^8^ CFU mL^−1^ of *S*. Typhimurium (strain SL1344) from Hangzhou Yinyuan Biotechnology Company. The body weight and survival of the rats were recorded, and a diarrhea score was assigned.

WIMT was performed in accordance with the protocols described by Li et al.^[^ ^28^ ^]^ and Kim et al.,^[^ ^63^ ^]^ with some modifications. WIMT refers to the simultaneous transplantation of microbiota from the jejunum, ileum, cecum, and colon. WIMT is more effective in facilitating engraftment of small intestinal microbiota than FMT.^[^ ^28^, ^74^ ^]^ Briefly, the contents of the jejunum, ileum, cecum, and colon were harvested from 8‐day‐old rats in an anaerobic chamber (LABIOPHY, Dalian, China) containing 10% CO_2_, 10% H_2_, and 80% N_2_. Then, 40 mL of PBS with 10% glycerol was added to 1 g of the contents, and the mixture was vortexed and passed through a 70‐µm filter to remove large particulates. The filtered mixture was centrifuged at 200 × *g* for 2 min, and the supernatant was divided into cryotubes and stored at –80 °C.

HCA (Merck, Cat#: 700159P) was administered at 100 mg kg^−1^ per day. GW4064 (MCE, Cat#: HY‐50108), BSH‐IN‐1 (MCE, HY‐135659), and GSK805 (MCE, HY‐12776) were administered at 50, 10, and 20 mg kg^−1^ per day, respectively. The *L. reuteri* strain (BNCC 186 563) was cultured in Murashige and Skoog medium (Hopebiol, Cat#: HB0384‐1) for 24 h at 37 °C, collected in the logarithmic phase (6 h) of growth, and then diluted to different concentrations for use.

Rats were sacrificed by CO_2_ inhalation, and the tissues were collected, fixed in 4% paraformaldehyde, and then stored at −80 °C for subsequent analyses.

### Hypoxyprobe

Hypoxyprobe (Cat#: HP1) was purchased from Hypoxyprobe, Inc. (Burlington, USA) and used in accordance with the manufacturer's instructions. Briefly, 60 mg kg^−1^ hypoxyprobe was intraperitoneally injected into rats, and the tissues were harvested after 1.5 h and subjected to immunofluorescence staining.

### RNA Isolation and Quantitative Real‐Time PCR

For LPLs and tissue, total RNA was extracted using the TRIzol reagent (Vazyme, Cat#: R401‐01). For sorted cells, total RNA was extracted using the PureLink RNA Micro Scale Kit (Thermo, Cat#: 12 183 016). Then, the concentration of RNA was detected on a NanoDropfi ND‐1000 Spectrophotometer (Thermo, Waltham, USA) and transcribed into cDNA using Reverse Transcriptase (ABclonal, Cat#: RK20433). mRNA levels were quantified through qPCR on a real‐time PCR system (Bio‐Rad, California, USA) using SYBR Green qPCR Master Mix (Vazyme, Cat#: Q321). The sequences of the primers are listed in Table S1 (Supporting Information).

### Intestinal Microbiome Sequencing

This technique was performed following a method previously described by Xia et al.^[^ ^75^ ^]^ Briefly, a QIAamp DNA Stool Mini Kit (QiagenLtd, Frankfurt, Germany) was used to extract total microbial genomic DNA from intestinal contents following the manufacturer's instructions. Amplicon libraries were sequenced on an Illumina MiSeq (Illumina, Santiago, USA) at Personalbio, Shanghai, China. Microbiome bioinformatics analysis was performed using QIIME 2 2019.4.^[^ ^76^ ^]^ The DADA2 plugin was used to filter the sequences for quality, denoise, merge, and remove chimeras.^[^ ^77^ ^]^ To assign taxonomic labels to the ASVs, the classify‐sklearn naïve Bayes taxonomy classifier was used in the feature‐classifier plugin^[^ ^78^ ^]^ to compare the reads to the Greengenes 13_8 99% operational taxonomic unit reference sequences.^[^ ^79^ ^]^ The functional potential of the intestinal microbiota was predicted using PICRUSt2.^[^ ^80^ ^]^

### Analysis of the Intestinal Microbiota

Chao1 values were used to assess alpha‐diversity and were calculated based on the relative abundance of each taxon. The Chao 1 index indicates a sample's diversity based on the observed and estimated number of species. Beta‐diversity was analyzed by PCoA based on Bray–Curtis dissimilarity index matrices. A random forest algorithm, modified from that described by Gao et al.,^[^ ^81^ ^]^ was used to model maturation of the intestinal microbiome, modified from that described by Gao et al.^[^ ^81^ ^]^ An unsupervised clustering algorithm, modified from that described by Zhou et al.,^[^ ^22^ ^]^ was used to separate mature and immature microbiomes. For the co‐occurrence network diagram, differences in abundance were analyzed by Spearman's rank correlation analysis, and ASVs were selected with prevalence in >30%. Co‐occurrence networks were constructed using data with correlation coefficients |r‐value| > 0.8 and *p* < 0.05. Gephi (v 0.10.1) was used for visualization and network analysis.

### Isolation of Intestinal LPLs and Flow Cytometry

Intestinal LPLs were isolated as previously described with some modifications.^[^ ^82^ ^]^ Briefly, the small intestine was dissected, and the fat and mesenteric tissues were removed. The intestines were dissected longitudinally, cut into several pieces, and then washed with cold Hanks’ balanced salt solution (Solarbio, Cat#: H1046). To remove epithelial cells, the intestines in HBSS were incubated with 2% FBS, 1 mm dithiothreitol (Servicebio, Cat#: GC205010), and 30 mm EDTA (Sanangon, Cat#: B540625) for 30 min at 37 °C while shaking at 200 rpm. The tissues were then digested with 50 µg mL^−1^ DNase I (Roche, Cat#: 10 104 159 001) and 300 U mL^−1^ collagenase VIII (Sigma, Cat#: C2139) in RPMI1640 medium (Servicebio, Cat#: G4533) for 30 min at 37 °C while shaking at 200 rpm. The digested tissues were homogenized by vigorous shaking and filtered through a 70 µm cell strainer. Subsequently, mononuclear cells were collected by centrifuging at 450 × *g* for 5 min. The cells were added to an 80% to 40% Percoll (Cytiva, Cat#: 365 237) gradient, centrifuged at 800 × *g* for 15 min at RT, and then harvested from the interphase.

For cytokine staining, cells were stimulated with Cell Stimulation Cocktail (plus protein transport inhibitors) (500×) (eBioscience, Cat#: 00‐4975‐93) for 5 h. The harvested cells were stained with Fixable Viability Stain (BD Pharmingen, Cat#:566 332) to differentiate between live and dead cells. After staining, the cells were blocked with an anti‐CD32 antibody. Subsequently, the cells were incubated with a mixture of antibodies to surface proteins, fixed and permeabilized using a Transcription Factor Buffer Set (BD Pharmingen, Cat#: 562 574), and then incubated with an intracellular flow cytometry antibody mixture. The panel of antibodies was selected based on previously published studies,^[^ ^83^, ^84^ ^]^ and the details of the antibodies used are shown in **Table** **1**. Analysis was performed on a CytoflexLX (Beckman, USA), and sorting was performed on a CytoFLEX SRT (Beckman, USA). FACS‐gating strategies for different immune cell populations are shown in Figure S1 (Supporting Information). For analysis, lymphocytes were identified as CD45^+^; T cells as CD45^+^CD3^+^CD45R^−^; B cells as CD45^+^CD3^−^CD45R^+^; monocytes as CD45^+^CD3^−^CD45R^−^; γδT cells as CD45^+^CD3^+^γδT^+^; Th17 cells as CD45^+^CD3^+^CD4^+^IL‐17A^+^; and ILC3s as CD45^+^CD3^−^CD45R^−^SIPR‐a^−^CD127^+^RORγt^+^. For sorting, γδT cells were identified as CD45^+^CD3^+^γδT^+^, and ILC3s were identified as CD45^+^CD3^−^CD45R^−^SIPR‐a^−^CD127^+^NK1.1^−^KLRG1^−^.

**Table 1:** Antibodies used in flow cytometry.

Marker	Channel	Cat#	Company	Clone	Refs.
CD32	FC blocking	550 271	BD Pharmingen	D34‐485	[85]
SIRP‐α	PE	MA517504	Thermo	OX41	[84]
CD3	PE	550 353	BD Pharmingen	1F4	[86]
CD45RA	PE	554 881	BD Pharmingen	HIS24	[87]
CD127	Alexa Fluor594	FAB8484T	R&D system	717 519	[83]
CD45	APC‐Cy7	561 586	BD Pharmingen	OX‐1	[88]
RORγt	APC	130‐123‐840	Miltenyi	REA278	[83]
IL‐22	PerCP/Cyanine5.5	516 411	BioLegend	Poly5164	[83]
IL‐17A	PE‐Cy7	25‐7177‐82	Thermo	eBio17B7	[89]
CD3	FITC	559 975	BD Pharmingen	G4.18	[90]
CD68	PE	130‐123‐757	Miltenyi	REA237	[91]
CD45RA	BV421	740 043	BD Pharmingen	OX‐33	[92]
γδ T	BV650	745 392	BD Pharmingen	V65	[93]
CD8	BV786	740 913	BD Pharmingen	OX‐8	[94]
CD4	APC	550 057	BD Pharmingen	OX‐35	[95]
NK1.1	BV605	744 051	BD Pharmingen	10/78	[96]
KLRG1	APC	sc‐32755	Santa Cruz Biotechnology	2F1	[97]

### Hematoxylin and Eosin (H&E) Staining and Immunofluorescence Staining

For H&E staining, intestinal sections were deparaffinized, rehydrated in water, and mounted on slides. Then, the slides were immersed in hematoxylin solution for 3–5 min and rinsed in water. Next, the sections were differentiated with acid alcohol, rinsed again, stained blue with an ammonia solution, washed in slowly running tap water, and then stained with eosin. Last, they were dehydrated and mounted with coverslips. Images of the sections were taken using a Panoramic SCAN (3DHISTECH CaseViewer, Budapest, Hungary). Cell morphology and histological scores were analyzed using CaseViewer software (3DHISTECH CaseViewer, Budapest, Hungary). The distance was measured from the apical side to the basal side of the crypts based on at least 10 intact and well‐oriented crypts in each sample. The histological score was assessed based on the degree of inflammation (1–2), lesion depth (1–4), and lesion extent (1–4).

For immunofluorescence staining, tissue sections were washed three times with PBS and then blocked with 5% BSA for 1 h at room temperature. Then, the PMDZ primary antibody diluent (1:100 in PBS) was added, and the sections were stored at 4 °C overnight. The primary antibody solution was removed the next day, and the sections were washed three times with PBS in the dark. Subsequently, DAPI staining solution (200 µL) was added, and the sections were incubated at room temperature for 20 min in the dark and then washed three times with PBS in the dark. To prepare the slides, 5 µL of an anti‐quenching mounting solution was added to each sample, coverslips were placed on the mounting solution, and the cover‐slipped slides were stored at 4 °C in the dark. Images were obtained after overnight observation under a laser confocal microscope.

### Bacterial Load Assay

To quantify the bacterial load, 50 mg of each sample in a sterile EP tube was resuspended with 0.5 mL of PBS containing 1% Triton X‐100 (Servicebio, Cat#: GC204003). This solution was then serially diluted 1:10 in PBS and plated onto bismuth sulfite agar (Huankai, Cat#: 02 7319). After incubation for 18 h at 37 °C, CFUs were counted.

### ELISA Analysis

Tissues were pulverized with a homogenizer, and the supernatants were collected for ELISA. The protein concentration of each sample was determined using a bicinchoninic acid kit. The concentrations of cytokines in the intestine were measured using rat IL‐22 (MM‐0670R1) and IL‐17A (MM‐70049R1) ELISA kits purchased from Jiangsu Meimian Industrial Co., Ltd.

### Molecular Docking Analysis

The FXR ligand‐binding domain crystal structure was downloaded from the RCSB Protein Data Bank (PDB ID: 5YXJ). The molecular structure of HCA was retrieved from PubChem Compound (CID: 92 805) and was prepared using SYBYL‐X 2.0, a molecular modeling software package that optimizes the geometry and 3D conformation of molecules for computational analysis. Molecular docking studies were performed using AutoDock Vina 1.2.2, a computational tool for protein–ligand docking that predicts the preferred binding position of a ligand with a protein. The binding interactions were visualized and edited using PyMOL, a molecular visualization system.

### Cell Culture, Transfection, and Treatment Conditions

HEK293T cells were cultured in high‐glucose Dulbecco's modified Eagle's medium (Hyclone, Cat#: SH30243.01) supplemented with 10% fetal bovine serum (NEWZERUM, Cat#: FBS‐S500) and 1% penicillin/streptomycin (Hyclone, Cat#: SV30010) at 37 °C in a 5% CO_2_ atmosphere. HEK293T cells were transfected with different vectors and exposed to different concentrations of HCA (Merck, Cat#: 700159P) and GW4064 (MCE, Cat#: HY‐50108) for 24 h for the luciferase reporter assay.

Freshly isolated intestinal LPLs were added to a 24‐well plate and cultured in RPMI 1640 medium (servicebio, Cat#: G4533) supplemented with 10% fetal bovine serum (NEWZERUM, Cat No: FBS‐S500), 1% penicillin/streptomycin (Hyclone, Cat#: SV30010), and Cell Stimulation Cocktail (plus protein transport inhibitors) (500×) (eBioscience, Cat#: 00‐4975‐93) at 37 °C in a 5% CO_2_ atmosphere, with different concentrations of HCA, for 16 h and then collected for analysis.

Twenty thousand of sorting ILC3s or γδT cells were to a 24‐well plate and cultured in RPMI 1640 medium (servicebio, Cat#: G4533) supplemented with 10% fetal bovine serum (NEWZERUM, Cat No: FBS‐S500), 1% penicillin/streptomycin (Hyclone, Cat#: SV30010), and at 37 °C in a 5% CO_2_ atmosphere, with 100 µm HCA (Merck, Cat#: 700159P), 10 µm β‐MCA (MCE, Cat#: HY‐114392), 10 µm GW4064 (MCE, Cat#: HY‐50108), for 16 h and then collected for analysis.

### Luciferase Reporter Assay

The pGL3‐Basic‐WTAP firefly luciferase reporter vector and versions of the plasmid containing different fragments of the WTAP promoter were constructed by Tsingke Biotechnology. The human NR4H1 (FXR) expression vector and human ASBT expression vector were purchased from GeneCopoeia. HEK293T cells were cultured and co‐transfected with the human FXR expression vector, human ASBT expression vector, pGL3‐Basic‐WTAP or pGL3‐Basic‐WTAP P1‐P4, deletion or mutation luciferase reporter vector, and Renilla luciferase control vector (Promega, Madison, WI) using Lipo8000 transfection reagent (Beyotime, Cat#: C0533). Luciferase assays were performed using the Dual‐Glo Luciferase Assay System (Promega, Cat#: E2920). Firefly and Renilla luciferase activities were measured using a Synergy2 instrument (BioTek, USA).

### Bile Acid Analysis

BA contents were detected using MetWare (http://www.metware.cn/) based on the AB Sciex QTRAP 6500 LC‐MS/MS platform. Briefly, samples (20 mg) were ground in a ball mill and extracted with 200 µL of methanol/acetonitrile (v/v = 2:8). Next, 10 µL of an internal standard solution (1 µg mL^−1^) was added to the extract to serve as an internal standard (IS) for quantification, and the samples were incubated at −20 °C for 10 min to precipitate protein. Next, the samples were centrifuged at 12000 r min^−1^ for 10 min at 4 °C, and the supernatant was transferred to clean plastic microtubes. The extracts were dried and then reconstituted in 100 µL of 50% methanol (V/V) for further analysis. The extracts were analyzed by LC‐ESI‐MS/MS. The relative quantities of each BA were calculated by dividing the amount of each BA by the total BA content.

### m^6^A Dot Blot

Dot blotting was performed as described previously. First, a denaturing solution was prepared consisting of 60 µL of 20 × SSC buffer (Biosharp, Cat#: BL164A) and 40 µL of 37% deionized formaldehyde. RNA and the denaturing solution were combined at a 1:1 ratio, and the mixture was incubated at 95 °C for 5 min. Next, the denatured samples were quickly spotted onto a nitrocellulose (NC) membrane and then cross‐linked under a 302 nm UV lamp for 6 min. Positive signals on the cross‐linked NC membrane were detected using a m^6^A‐specific antibody (Abclonal, Cat#: A19841). Secondary anti‐rabbit IgG‐HRP antibody (1:5000, Abclonal, Cat#: AS014) was used to detect primary antibodies. Binding was detected using an enhanced chemiluminescence detection kit, and densitometry was performed using Image‐J software (Bethesda, Maryland, USA).

### RNA Stability Assay

Sorted cells were cultured in RPMI 1640 medium (Servicebio, Cat#: G4533) supplemented with 10% fetal bovine serum (NEWZERUM, Cat No: FBS‐S500) and 1% penicillin/streptomycin (Hyclone, Cat#: SV30010) at 37 °C in a 5% CO_2_ atmosphere. Actinomycin D (MCE, Cat No: HY‐17559) was added to a final concentration of 1 µm, and cells were harvested 5 h later. The RNA was extracted and subjected to RT‐qPCR.

### SELECT Detection Assay

An Epi‐SELECT m^6^A site identification kit (Epibiotek, Cat#: R202106M‐01) was purchased from Guangzhou Epibiotek Co., Ltd. and used as described by Xiao et al.^[^ ^98^ ^]^ Briefly, 20 ng RNA was mixed with a forward primer, a reverse primer, and dNTPs in 1 × CutSmart buffer and then annealed and extended in a thermal cycler (Bio‐Rad, California, USA). Subsequently, a mixture of SELECT DNA polymerase, SELECT ligase, and ATP was added to the former mixture, and single‐base extension was carried out in a thermal cycler (Bio‐Rad, California, USA). Last, qPCR was performed on a real‐time PCR system (Bio‐Rad, California, USA) using SYBR Green qPCR Master Mix (Vazyme, Cat#: Q321). The primer sequences for the specific m^6^A sites in *Rorc* were as follows: Rorc_up_probe: tagccagtaccgtagtgcgtgCTTCTGGGTGCTTGCCACCAG, Rorc_down_probe: 5phos/ CTCTGAGCTAGATCCATCTCCCCCcagaggctgagtcgctgcat.

### Statistical Analysis

Data were analyzed using GraphPad Prism (v 9.0.0) and Microsoft Excel (2023), flowjo (v 10.4), and expressed as the means ± standard error of the mean (SEM). First, the normality of the data distribution was assessed using the Shapiro−Wilk test. For data following a normal distribution, a *t*‐test was used for pairwise comparisons. For non‐normally distributed data, the Mann−Whitney U‐test was applied, as this non‐parametric test does not require assumptions about the distribution of the data and was robust for small sample sizes. Data from different age groups were analyzed using one‐way ANOVA, followed by Tukey's multiple comparisons test for post hoc adjustments. Statistical significance was defined as *P* < 0.05. Graphical representations were prepared using GraphPad Prism version 9.0.0 software (San Diego, California, USA), imageGP.^[^ ^99^ ^]^ Correlation analysis was performed using Spearman's rank test correlation analysis, which is suitable for ordinal or non‐normally distributed data. The significance of survival dissimilarity was calculated by the Log‐rank (Mantel–Cox) test. The significance of body weight variation dissimilarity was calculated by two‐way ANOVA followed by pairwise comparisons adjusted using Tukey's multiple comparisons test. The graphical abstract was prepared using BioRender.

## Conflict of Interest

The authors declare no conflict of interest.

## Author Contributions

Z.P.Y., P.J., and H.K.W. conceived the project and designed the study. Z.P.Y., Z.Y.L, Y.J.Y, M.Z., N.G. X.C.L, and X.R.L performed the experiments and analyzed the data. Z.P.Y., P.J., and H.K.W. wrote and revised the manuscript. All the authors edited the manuscript and approved the final manuscript.

## Supporting information



Supporting Information

Supplemental Table 1

## Data Availability

Data will be publicly available upon acceptance. The data that support the findings of this study are openly available in PRJNA1202948.

## References

[1] T. R. Kollmann , A. Marchant , S. S. Way , Science 2020, 368, 612. 32381718 10.1126/science.aaz9447 PMC7734703

[2] T. W. BAND, Mortality rate, under‐5 (per 1000 live births) 2023.

[3] I. I. Ivanov , L. Frutos Rde , N. Manel , K. Yoshinaga , D. B. Rifkin , R. B. Sartor , B. B. Finlay , D. R. Littman , Cell Host Microbe 2008, 4, 337. 18854238 10.1016/j.chom.2008.09.009 PMC2597589

[4] X. Qi , J. Qiu , J. Chang , Y. Ji , Q. Yang , G. Cui , L. Sun , Q. Chai , J. Qin , J. Qiu , Mucosal Immunol. 2021, 14, 38. 32612160 10.1038/s41385-020-0317-3 PMC7790751

[5] G. Eberl , Mucosal Immunol. 2017, 10, 27. 27706126 10.1038/mi.2016.86

[6] W. Ren , Y. Liao , X. Ding , Y. Jiang , J. Yan , Y. Xia , B. Tan , Z. Lin , J. Duan , X. Jia , G. Yang , J. Deng , C. Zhu , P. R. Hardwidge , J. Li , G. Zhu , Y. Yin , Mucosal Immunol. 2019, 12, 531. 30523310 10.1038/s41385-018-0111-7

[7] L. Xiong , E. Y. Helm , J. W. Dean , N. Sun , F. R. Jimenez‐Rondan , L. Zhou , Nat. Immunol. 2023, 24, 1671. 37709985 10.1038/s41590-023-01612-z PMC11256193

[8] Y. S. Chen , I. B. Chen , G. Pham , T. Y. Shao , H. Bangar , S. S. Way , D. B. Haslam , J. Clin. Invest. 2020, 130, 2377. 31990686 10.1172/JCI127242 PMC7190913

[9] X. Niu , S. Daniel , D. Kumar , E. Y. Ding , R. C. Savani , A. Y. Koh , J. Mirpuri , Sci. Rep. 2020, 10, 1. 32737397 10.1038/s41598-020-69797-z PMC7395748

[10] S. Jin , D. Zhao , C. Cai , D. Song , J. Shen , A. Xu , Y. Qiao , Z. Ran , Q. Zheng , Sci. Rep. 2017, 7, 43662. 28272549 10.1038/srep43662 PMC5341569

[11] N. Satoh‐Takayama , C. A. Vosshenrich , S. Lesjean‐Pottier , S. Sawa , M. Lochner , F. Rattis , J. J. Mention , K. Thiam , N. Cerf‐Bensussan , O. Mandelboim , G. Eberl , J. P. Di Santo , Immunity 2008, 29, 958. 19084435 10.1016/j.immuni.2008.11.001

[12] L. Dupraz , A. Magniez , N. Rolhion , M. L. Richard , G. Da Costa , S. Touch , C. Mayeur , J. Planchais , A. Agus , C. Danne , C. Michaudel , M. Spatz , F. Trottein , P. Langella , H. Sokol , M. L. Michel , Cell Rep. 2021, 36, 109332. 34233192 10.1016/j.celrep.2021.109332

[13] E. Chun , S. Lavoie , D. Fonseca‐Pereira , S. Bae , M. Michaud , H. R. Hoveyda , G. L. Fraser , C. A. Gallini Comeau , J. N. Glickman , M. H. Fuller , B. T. Layden , W. S. Garrett , Immunity 2019, 51, 871. 31628054 10.1016/j.immuni.2019.09.014 PMC6901086

[14] B. Stockinger , K. Shah , E. Wincent , Nat. Rev. Gastroenterol. Hepatol. 2021, 18, 559. 33742166 10.1038/s41575-021-00430-8 PMC7611426

[15] V. Woo , T. Alenghat , Gut Microbes 2022, 14, 2 022 407. 10.1080/19490976.2021.2022407 PMC8744890 35000562

[16] X. S. Zhang , Y. S. Yin , J. Wang , T. Battaglia , K. Krautkramer , W. V. Li , J. Li , M. Brown , M. Zhang , M. H. Badri , A. J. S. Armstrong , C. M. Strauch , Z. Wang , I. Nemet , N. Altomare , J. C. Devlin , L. He , J. T. Morton , J. A. Chalk , K. Needles , V. Liao , J. Mount , H. Li , K. V. Ruggles , R. A. Bonneau , M. G. Dominguez‐Bello , F. Bäckhed , S. L. Hazen , M. J. Blaser , Cell Host Microbe 2021, 29, 1249. 34289377 10.1016/j.chom.2021.06.014 PMC8370265

[17] S. Jabs , A. Biton , C. Bécavin , M. A. Nahori , A. Ghozlane , A. Pagliuso , G. Spanò , V. Guérineau , D. Touboul , Q. Giai Gianetto , T. Chaze , M. Matondo , M. A. Dillies , P. Cossart , Nat. Commun. 2020, 11, 1344. 32165618 10.1038/s41467-020-15126-x PMC7067863

[18] X. Wang , Y. Li , W. Chen , H. Shi , A. M. Eren , A. Morozov , C. He , G. Z. Luo , T. Pan , Cell Res. 2019, 29, 167. 30559439 10.1038/s41422-018-0127-2 PMC6355850

[19] R. Zhuo , M. Xu , X. Wang , B. Zhou , X. Wu , V. Leone , E. B. Chang , X. Zhong , Wiley Interdiscip. Rev. RNA 2022, 13, 1725. 10.1002/wrna.1725 35301791

[20] C. Liu , Z. Yang , R. Li , Y. Wu , M. Chi , S. Gao , X. Sun , X. Meng , B. Wang , J. Transl. Med. 2021, 19, 251. 34103054 10.1186/s12967-021-02918-y PMC8186046

[21] L. Shenhav , K. Fehr , M. E. Reyna , C. Petersen , D. L. Y. Dai , R. Dai , V. Breton , L. Rossi , M. Smieja , E. Simons , M. A. Silverman , M. Levy , L. Bode , C. J. Field , J. S. Marshall , T. J. Moraes , P. J. Mandhane , S. E. Turvey , P. Subbarao , M. G. Surette , M. B. Azad , Cell 2024, 187, 5431. 39303691 10.1016/j.cell.2024.07.022 PMC11531244

[22] L. Zhou , W. Qiu , J. Wang , A. Zhao , C. Zhou , T. Sun , Z. Xiong , P. Cao , W. Shen , J. Chen , X. Lai , L. H. Zhao , Y. Wu , M. Li , F. Qiu , Y. Yu , Z. Z. Xu , H. Zhou , W. Jia , Y. Liao , R. Retnakaran , D. Krewski , S. W. Wen , J. C. Clemente , T. Chen , R. H. Xie , Y. He , Cell Host Microbe 2023, 31, 1232. 37327780 10.1016/j.chom.2023.05.022

[23] J. Wang , M. G. Dominguez‐Bello , Nat. Microbiol. 2020, 5, 785. 32467622 10.1038/s41564-020-0734-9

[24] C. J. Kelly , L. Zheng , E. L. Campbell , B. Saeedi , C. C. Scholz , A. J. Bayless , K. E. Wilson , L. E. Glover , D. J. Kominsky , A. Magnuson , T. L. Weir , S. F. Ehrentraut , C. Pickel , K. A. Kuhn , J. M. Lanis , V. Nguyen , C. T. Taylor , S. P. Colgan , Cell Host Microbe 2015, 17, 662. 25865369 10.1016/j.chom.2015.03.005 PMC4433427

[25] Y. Liang , X. Yao , Z. Meng , J. Lan , Y. Qiu , C. Cen , Y. Feng , BMC Microbiol. 2024, 24, 82. 38461289 10.1186/s12866-024-03234-3 PMC10924324

[26] A. Kanhere , A. Hertweck , U. Bhatia , M. R. Gökmen , E. Perucha , I. Jackson , G. M. Lord , R. G. Jenner , Nat. Commun. 2012, 3, 1268. 23232398 10.1038/ncomms2260 PMC3535338

[27] P. Pais , V. Almeida , M. Yılmaz , M. C. Teixeira , J. Fungi (Basel) 2020, 6, 78. 32512834 10.3390/jof6020078 PMC7344949

[28] N. Li , B. Zuo , S. Huang , B. Zeng , D. Han , T. Li , T. Liu , Z. Wu , H. Wei , J. Zhao , J. Wang , Microbiome 2020, 8, 161. 33208178 10.1186/s40168-020-00917-7 PMC7677849

[29] X. Zheng , T. Chen , R. Jiang , A. Zhao , Q. Wu , J. Kuang , D. Sun , Z. Ren , M. Li , M. Zhao , S. Wang , Y. Bao , H. Li , C. Hu , B. Dong , D. Li , J. Wu , J. Xia , X. Wang , K. Lan , C. Rajani , G. Xie , A. Lu , W. Jia , C. Jiang , W. Jia , Cell Metab. 2021, 33, 791. 33338411 10.1016/j.cmet.2020.11.017

[30] T. Fu , Y. Li , T. G. Oh , F. Cayabyab , N. He , Q. Tang , S. Coulter , M. Truitt , P. Medina , M. He , R. T. Yu , A. Atkins , Y. Zheng , C. Liddle , M. Downes , R. M. Evans , Proc. Natl. Acad. Sci. USA 2022, 119, 2213041119. 10.1073/pnas.2213041119 PMC9907109 36508655

[31] C. Jiang , C. Xie , Y. Lv , J. Li , K. W. Krausz , J. Shi , C. N. Brocker , D. Desai , S. G. Amin , W. H. Bisson , Y. Liu , O. Gavrilova , A. D. Patterson , F. J. Gonzalez , Nat. Commun. 2015, 6, 10166. 26670557 10.1038/ncomms10166 PMC4682112

[32] L. Gan , Y. Zhao , Y. Fu , Q. Chen , Biomed. Pharmacother. 2023, 160, 114343. 36758318 10.1016/j.biopha.2023.114343

[33] Z. Shulman , N. Stern‐Ginossar , Nat. Immunol. 2020, 21, 501. 32284591 10.1038/s41590-020-0650-4

[34] Z. Yang , X. Liu , Y. Wu , J. Peng , H. Wei , Front. Immunol. 2022, 13, 936 300. 10.3389/fimmu.2022.936300 PMC9344006 35928828

[35] H. Stepanova , M. Scheirichova , J. Matiasovic , K. Hlavova , M. Sinkora , K. Stepanova , M. Faldyna , Front. Immunol. 2023, 14, 1 214 444. 10.3389/fimmu.2023.1214444 PMC10548118 37799720

[36] G. F. Sonnenberg , D. Artis , Immunity 2012, 37, 601. 23084357 10.1016/j.immuni.2012.10.003 PMC3495160

[37] Ivanov, I. I. , K. Atarashi , N. Manel , E. L. Brodie , T. Shima , U. Karaoz , D. Wei , K. C. Goldfarb , C. A. Santee , S. V. Lynch , T. Tanoue , A. Imaoka , K. Itoh , K. Takeda , Y. Umesaki , K. Honda , D. R. Littman , Cell 2009, 139, 485. 19836068 10.1016/j.cell.2009.09.033 PMC2796826

[38] E. T. Grant , M. Boudaud , A. Muller , A. J. Macpherson , M. S. Desai , EMBO Mol. Med. 2023, 15, 17241. 10.15252/emmm.202217241 PMC10405054 37278126

[39] C. Campbell , P. T. McKenney , D. Konstantinovsky , O. I. Isaeva , M. Schizas , J. Verter , C. Mai , W. B. Jin , C. J. Guo , S. Violante , R. J. Ramos , J. R. Cross , K. Kadaveru , J. Hambor , A. Y. Rudensky , Nature 2020, 581, 475. 32461639 10.1038/s41586-020-2193-0 PMC7540721

[40] M. Arifuzzaman , T. H. Won , T. T. Li , H. Yano , S. Digumarthi , A. F. Heras , W. Zhang , C. N. Parkhurst , S. Kashyap , W. B. Jin , G. G. Putzel , A. M. Tsou , C. Chu , Q. Wei , A. Grier , S. Worgall , C. J. Guo , F. C. Schroeder , D. Artis , Nature 2022, 611, 578. 36323778 10.1038/s41586-022-05380-y PMC10576985

[41] G. Sorrentino , A. Perino , E. Yildiz , G. El Alam , M. Bou Sleiman , A. Gioiello , R. Pellicciari , K. Schoonjans , Gastroenterology 2020, 159, 956. 32485177 10.1053/j.gastro.2020.05.067

[42] M. M. Thibaut , L. B. Bindels , Trends Mol. Med. 2022, 28, 223. 35074252 10.1016/j.molmed.2021.12.006

[43] X. Qi , C. Yun , L. Sun , J. Xia , Q. Wu , Y. Wang , L. Wang , Y. Zhang , X. Liang , L. Wang , F. J. Gonzalez , A. D. Patterson , H. Liu , L. Mu , Z. Zhou , Y. Zhao , R. Li , P. Liu , C. Zhong , Y. Pang , C. Jiang , J. Qiao , Nat. Med. 2019, 25, 1225. 31332392 10.1038/s41591-019-0509-0 PMC7376369

[44] E. M. Eshleman , T. Rice , C. Potter , A. Waddell , S. Hashimoto‐Hill , V. Woo , S. Field , L. Engleman , H. W. Lim , M. A. Schumacher , M. R. Frey , L. A. Denson , F. D. Finkelman , T. Alenghat , Immunity 2024, 57, 319. 38295798 10.1016/j.immuni.2024.01.002 PMC10901458

[45] F. Zou , Y. Qiu , Y. Huang , H. Zou , X. Cheng , Q. Niu , A. Luo , J. Sun , Cell Death Dis. 2021, 12, 582. 34099635 10.1038/s41419-021-03880-9 PMC8184914

[46] F. Hao , M. Tian , X. Zhang , X. Jin , Y. Jiang , X. Sun , Y. Wang , P. Peng , J. Liu , C. Xia , Y. Feng , M. Wei , Proc. Natl. Acad. Sci. USA 2021, 118, 2014681118. 10.1073/pnas.2014681118 PMC8179238 34035164

[47] W. Yang , T. Yu , X. Huang , A. J. Bilotta , L. Xu , Y. Lu , J. Sun , F. Pan , J. Zhou , W. Zhang , S. Yao , C. L. Maynard , N. Singh , S. M. Dann , Z. Liu , Y. Cong , Nat. Commun. 2020, 11, 4457. 32901017 10.1038/s41467-020-18262-6 PMC7478978

[48] S. Guo , F. Chen , L. Li , S. Dou , Q. Li , Y. Huang , Z. Li , W. Liu , G. Zhang , J. Adv. Res. 2023, 61, 165. 37619934 10.1016/j.jare.2023.08.014 PMC11258656

[49] B. Liu , N. Liu , X. Zhu , L. Yang , B. Ye , H. Li , P. Zhu , T. Lu , Y. Tian , Z. Fan , Cell Mol. Immunol. 2021, 18, 1412. 33911218 10.1038/s41423-021-00680-1 PMC8166869

[50] M. H. Foley , S. O'Flaherty , G. Allen , A. J. Rivera , A. K. Stewart , R. Barrangou , C. M. Theriot , Proc. Natl. Acad. Sci. USA 2021, 118, 2017709118. 10.1073/pnas.2017709118 PMC8017965 33526676

[51] A. B. Larabi , H. L. P. Masson , A. J. Bäumler , Gut Microbes 2023, 15, 2 172 671. 10.1080/19490976.2023.2172671 PMC9904317 36740850

[52] S. L. Collins , J. G. Stine , J. E. Bisanz , C. D. Okafor , A. D. Patterson , Nat. Rev. Microbiol. 2023, 21, 236. 36253479 10.1038/s41579-022-00805-x PMC12536349

[53] X. Ye , D. Huang , Z. Dong , X. Wang , M. Ning , J. Xia , S. Shen , S. Wu , Y. Shi , J. Wang , X. Wan , Microbiol. Spectr. 2022, 10, 0051822. 10.1128/spectrum.00518-22 PMC9603329 36036629

[54] C. Hu , B. Xu , X. Wang , W. H. Wan , J. Lu , D. Kong , Y. Jin , W. You , H. Sun , X. Mu , D. Feng , Y. Chen , Hepatology 2023, 77, 48. 35262957 10.1002/hep.32449 PMC9970019

[55] T. L. Montgomery , K. Eckstrom , K. H. Lile , S. Caldwell , E. R. Heney , K. G. Lahue , A. D'Alessandro , M. J. Wargo , D. N. Krementsov , Microbiome 2022, 10, 198. 36419205 10.1186/s40168-022-01408-7 PMC9685921

[56] T. Wang , N. Zheng , Q. Luo , L. Jiang , B. He , X. Yuan , L. Shen , Front Immunol 2019, 10, 1235. 31214189 10.3389/fimmu.2019.01235 PMC6558076

[57] Q. Mu , B. K. Swartwout , M. Edwards , J. Zhu , G. Lee , K. Eden , X. Cabana‐Puig , D. K. McDaniel , J. Mao , L. Abdelhamid , R. M. Brock , I. C. Allen , C. M. Reilly , X. M. Luo , Proc. Natl. Acad. Sci. USA 2021, 118, 2015691118. 10.1073/pnas.2015691118 PMC7936341 33619092

[58] I. Vacca , Nat. Rev. Microbiol. 2017, 15, 574.

[59] F. Rivera‐Chávez , C. A. Lopez , A. J. Bäumler , Free Radical Biol. Med. 2017, 105, 93. 27677568 10.1016/j.freeradbiomed.2016.09.022

[60] S. A. Cevallos , J. Y. Lee , E. M. Velazquez , N. J. Foegeding , C. D. Shelton , C. R. Tiffany , B. H. Parry , A. R. Stull‐Lane , E. E. Olsan , H. P. Savage , H. Nguyen , S. S. Ghanaat , A. J. Byndloss , I. O. Agu , R. M. Tsolis , M. X. Byndloss , A. J. Bäumler , mBio 2021, 12, 3227. 10.1128/mBio.03227-20 PMC7845635 33468700

[61] M. X. Byndloss , E. E. Olsan , F. Rivera‐Chávez , C. R. Tiffany , S. A. Cevallos , K. L. Lokken , T. P. Torres , A. J. Byndloss , F. Faber , Y. Gao , Y. Litvak , C. A. Lopez , G. Xu , E. Napoli , C. Giulivi , R. M. Tsolis , A. Revzin , C. B. Lebrilla , A. J. Bäumler , Science 2017, 357, 570. 28798125 10.1126/science.aam9949 PMC5642957

[62] Y. Litvak , K. K. Z. Mon , H. Nguyen , G. Chanthavixay , M. Liou , E. M. Velazquez , L. Kutter , M. A. Alcantara , M. X. Byndloss , C. R. Tiffany , G. T. Walker , F. Faber , Y. Zhu , D. N. Bronner , A. J. Byndloss , R. M. Tsolis , H. Zhou , A. J. Bäumler , Cell Host Microbe 2019, 25, 128. 30629913 10.1016/j.chom.2018.12.003 PMC12036633

[63] Y. G. Kim , K. Sakamoto , S. U. Seo , J. M. Pickard , M. G. Gillilland 3rd , N. A. Pudlo , M. Hoostal , X. Li , T. D. Wang , T. Feehley , A. T. Stefka , T. M. Schmidt , E. C. Martens , S. Fukuda , N. Inohara , C. R. Nagler , G. Núñez , Science 2017, 356, 315. 28428425 10.1126/science.aag2029 PMC6082366

[64] J. R. Singer , E. G. Blosser , C. L. Zindl , D. J. Silberger , S. Conlan , V. A. Laufer , D. DiToro , C. Deming , R. Kumar , C. D. Morrow , J. A. Segre , M. J. Gray , D. A. Randolph , C. T. Weaver , Nat. Med. 2019, 25, 1772. 31700190 10.1038/s41591-019-0640-y PMC7250008

[65] C. Rao , K. Z. Coyte , W. Bainter , R. S. Geha , C. R. Martin , S. Rakoff‐Nahoum , Nature 2021, 591, 633. 33627867 10.1038/s41586-021-03241-8 PMC7990694

[66] M. J. Slifierz , R. M. Friendship , J. S. Weese , BMC Microbiol. 2015, 15, 184. 26391877 10.1186/s12866-015-0512-7 PMC4578254

[67] N. Van Best , U. Rolle‐Kampczyk , F. Schaap , M. Basic , S. O. Damink , A. Bleich , P. Savelkoul , M. Von Bergen , J. Penders , M. Hornef , Nat. Commun. 2020, 11, 3692. 32703946 10.1038/s41467-020-17183-8 PMC7378201

[68] R. Singhal , Y. M. Shah , J. Biol. Chem. 2020, 295, 10493. 32503843 10.1074/jbc.REV120.011188 PMC7383395

[69] J. L. Fachi , L. P. Pral , J. A. C. Dos Santos , A. C. Codo , S. de Oliveira , J. S. Felipe , F. F. F. Zambom , N. O. S. Câmara , P. Vieira , M. Colonna , M. A. R. Vinolo , Mucosal Immunol. 2021, 14, 828. 33446906 10.1038/s41385-020-00371-6 PMC8221997

[70] F. Annunziato , C. Romagnani , S. Romagnani , J. Allergy Clin. Immunol. 2015, 135, 626. 25528359 10.1016/j.jaci.2014.11.001

[71] K. Atarashi , T. Tanoue , M. Ando , N. Kamada , Y. Nagano , S. Narushima , W. Suda , A. Imaoka , H. Setoyama , T. Nagamori , E. Ishikawa , T. Shima , T. Hara , S. Kado , T. Jinnohara , H. Ohno , T. Kondo , K. Toyooka , E. Watanabe , S. Yokoyama , S. Tokoro , H. Mori , Y. Noguchi , H. Morita , I. I. Ivanov , T. Sugiyama , G. Nuñez , J. G. Camp , M. Hattori , Y. Umesaki , et al., Cell 2015, 163, 367. 26411289 10.1016/j.cell.2015.08.058 PMC4765954

[72] I. Godinez , M. Raffatellu , H. Chu , T. A. Paixão , T. Haneda , R. L. Santos , C. L. Bevins , R. M. Tsolis , A. J. Bäumler , Infect. Immun. 2009, 77, 387. 18955477 10.1128/IAI.00933-08 PMC2612270

[73] M. Raffatellu , R. L. Santos , D. E. Verhoeven , M. D. George , R. P. Wilson , S. E. Winter , I. Godinez , S. Sankaran , T. A. Paixao , M. A. Gordon , J. K. Kolls , S. Dandekar , A. J. Bäumler , Nat. Med. 2008, 14, 421. 18376406 10.1038/nm1743 PMC2901863

[74] Y. Yang , J. He , Y. Wang , L. Liang , Z. Zhang , X. Tan , S. Tao , Z. Wu , M. Dong , J. Zheng , H. Zhang , S. Feng , W. Cheng , Q. Chen , H. Wei , Front. Immunol. 2023, 14, 1143526. 37234168 10.3389/fimmu.2023.1143526 PMC10206398

[75] X. Xia , J. J. Ni , S. N. Yin , Z. P. Yang , H. N. Jiang , C. Wang , J. Peng , H. K. Wei , X. Y. Wang , Front. Microbiol. 2021, 12, 686648. 34512565 10.3389/fmicb.2021.686648 PMC8424189

[76] E. Bolyen , J. R. Rideout , M. R. Dillon , N. Bokulich , C. C. Abnet , G. A. Al‐Ghalith , H. Alexander , E. J. Alm , M. Arumugam , F. Asnicar , Y. Bai , J. E. Bisanz , K. Bittinger , A. Brejnrod , C. J. Brislawn , C. T. Brown , B. J. Callahan , A. M. Caraballo‐Rodriguez , J. Chase , E. K. Cope , R. Da Silva , C. Diener , P. C. Dorrestein , G. M. Douglas , D. M. Durall , C. Duvallet , C. F. Edwardson , M. Ernst , M. Estaki , J. Fouquier , et al., Nat. Biotechnol. 2019, 37, 852. 31341288 10.1038/s41587-019-0209-9 PMC7015180

[77] M. Martin , EMBnet J. 2011, 17, 1.

[78] N. A. Bokulich , B. D. Kaehler , J. R. Rideout , M. Dillon , E. Bolyen , R. Knight , G. A. Huttley , J. G. Caporaso , Microbiome 2018, 6, 17. 29773078 10.1186/s40168-018-0470-z PMC5956843

[79] D. McDonald , M. N. Price , J. Goodrich , E. P. Nawrocki , T. Z. DeSantis , A. Probst , G. L. Andersen , R. Knight , P. Hugenholtz , Isme Journal 2012, 6, 610. 22134646 10.1038/ismej.2011.139 PMC3280142

[80] G. M. Douglas , V. J. Maffei , J. R. Zaneveld , S. N. Yurgel , J. R. Brown , C. M. Taylor , C. Huttenhower , M. G. I. Langille , Nat. Biotechnol. 2020, 38, 685. 32483366 10.1038/s41587-020-0548-6 PMC7365738

[81] P. Gao , C. Ma , Z. Sun , L. Wang , S. Huang , X. Su , J. Xu , H. Zhang , Microbiome 2017, 5, 91. 28768551 10.1186/s40168-017-0315-1 PMC5541433

[82] J. J. Zhang , L. Shen , Bio‐101 2019, 9, 1010327.

[83] A. Abidi , T. Laurent , G. Bériou , L. Bouchet‐Delbos , C. Fourgeux , C. Louvet , R. Triki‐Marrakchi , J. Poschmann , R. Josien , J. Martin , Front. Immunol. 2020, 11, 255. 32140157 10.3389/fimmu.2020.00255 PMC7043102

[84] A. Sudworth , F. M. Segers , B. Yilmaz , N. C. Guslund , A. J. Macpherson , E. Dissen , S. W. Qiao , M. Inngjerdingen , Eur. J. Immunol. 2022, 52, 717. 35099074 10.1002/eji.202149639

[85] Y. Shimamura , K. Furuhashi , A. Tanaka , M. Karasawa , T. Nozaki , S. Komatsu , K. Watanabe , A. Shimizu , S. Minatoguchi , M. Matsuyama , Y. Sawa , N. Tsuboi , T. Ishimoto , H. I. Suzuki , S. Maruyama , Commun. Biol. 2022, 5, 753. 35902687 10.1038/s42003-022-03712-2 PMC9334610

[86] D. Pouyabahar , S. W. Chung , O. I. Pezzutti , C. T. Perciani , X. Wang , X. Z. Ma , C. Jiang , D. Camat , T. Chung , M. Sekhon , J. Manuel , X. C. Chen , I. D. McGilvray , S. A. MacParland , G. D. Bader , iScience 2023, 26, 108213. 38026201 10.1016/j.isci.2023.108213 PMC10651689

[87] A. Capuz , S. Osien , M. A. Karnoub , S. Aboulouard , E. Laurent , E. Coyaud , A. Raffo‐Romero , M. Duhamel , A. Bonnefond , M. Derhourhi , M. Trerotola , I. El Yazidi‐Belkoura , D. Devos , M. Zilkova , F. Kobeissy , F. Vanden Abeele , I. Fournier , D. Cizkova , F. Rodet , M. Salzet , Cell Death Dis. 2023, 14, 237. 37015912 10.1038/s41419-023-05737-9 PMC10073301

[88] P. Bisaccia , F. Magarotto , S. D'Agostino , A. Dedja , S. Barbon , D. Guidolin , C. Liboni , R. Angioni , G. De Lazzari , F. Caicci , A. Viola , M. Jurga , G. Kundrotas , D. Stevens , D. Mancuso , E. Gramegna , B. Seitaj , R. Kashyap , B. De Vos , V. Macchi , E. Baraldi , A. Porzionato , R. De Caro , M. Muraca , M. Pozzobon , Stem Cells Transl. Med. 2024, 13, 43. 37963808 10.1093/stcltm/szad070 PMC10785219

[89] T. Du , C. L. Yang , M. R. Ge , Y. Liu , P. Zhang , H. Li , X. L. Li , T. Li , Y. D. Liu , Y. C. Dou , B. Yang , R. S. Duan , Front. Immunol. 2020, 11, 1603. 32793234 10.3389/fimmu.2020.01603 PMC7390899

[90] S. Hino , T. Mizushima , K. Kaneko , E. Kawai , T. Kondo , T. Genda , T. Yamada , K. Hase , N. Nishimura , T. Morita , J. Nutr. 2020, 150, 2656. 32286621 10.1093/jn/nxaa097

[91] H. Li , T. Zhao , Z. Yuan , T. Gao , Y. Yang , R. Li , Q. Tian , P. Tang , Q. Guo , L. Zhang , Bioact. Mater. 2024, 41, 61. 39104774 10.1016/j.bioactmat.2024.06.037 PMC11299526

[92] H. Yanai , C. Dunn , B. Park , C. Coletta , R. A. McDevitt , T. McNeely , M. Leone , R. P. Wersto , K. A. Perdue , I. Beerman , Elife 2022, 11, 76808. 10.7554/eLife.76808 PMC9150891 35507394

[93] P. Kühnlein , J. H. Park , T. Herrmann , A. Elbe , T. Hünig , J. Immunol. 1994, 153, 979. 8027567

[94] R. J. Brideau , P. B. Carter , W. R. McMaster , D. W. Mason , A. F. Williams , Eur. J. Immunol. 1980, 10, 609. 6967416 10.1002/eji.1830100807

[95] B. Sun , G. Li , L. Guo , N. Yin , H. Huang , X. Wu , R. Huang , M. Feng , iScience 2021, 24, 103 101. 10.1016/j.isci.2021.103101 PMC8479697 34622156

[96] H. Obara , K. Nagasaki , C. L. Hsieh , Y. Ogura , C. O. Esquivel , O. M. Martinez , S. M. Krams , Am. J. Transplant. 2005, 5, 2094. 16095488 10.1111/j.1600-6143.2005.00995.x PMC1473982

[97] L. Corral , T. Hanke , R. E. Vance , D. Cado , D. H. Raulet , Eur. J. Immunol. 2000, 30, 920. 10741410 10.1002/1521-4141(200003)30:3<920::AID-IMMU920>3.0.CO;2-P

[98] Y. Xiao , Y. Wang , Q. Tang , L. Wei , X. Zhang , G. Jia , Angew. Chem., Int. Ed. Engl. 2018, 57, 15995. 30345651 10.1002/anie.201807942

[99] T. Chen , Y.‐X. Liu , L. Huang , iMeta 2022, 1, 5. 10.1002/imt2.5 PMC10989750 38867732

