# Supplementary material for: Gut Microbiota‐Derived Hyocholic Acid Enhances Type 3 Immunity and Protects Against Salmonella enterica Serovar Typhimurium in Neonatal Rats
Source: Adv Sci (Weinh). 2024 Dec 31;12(10):2412071. doi: 10.1002/advs.202412071 (PMC11905087; doi:10.1002/advs.202412071)
Supplement: Supplementary file 1 — Supporting Information [file ADVS-12-2412071-s002.docx]

**
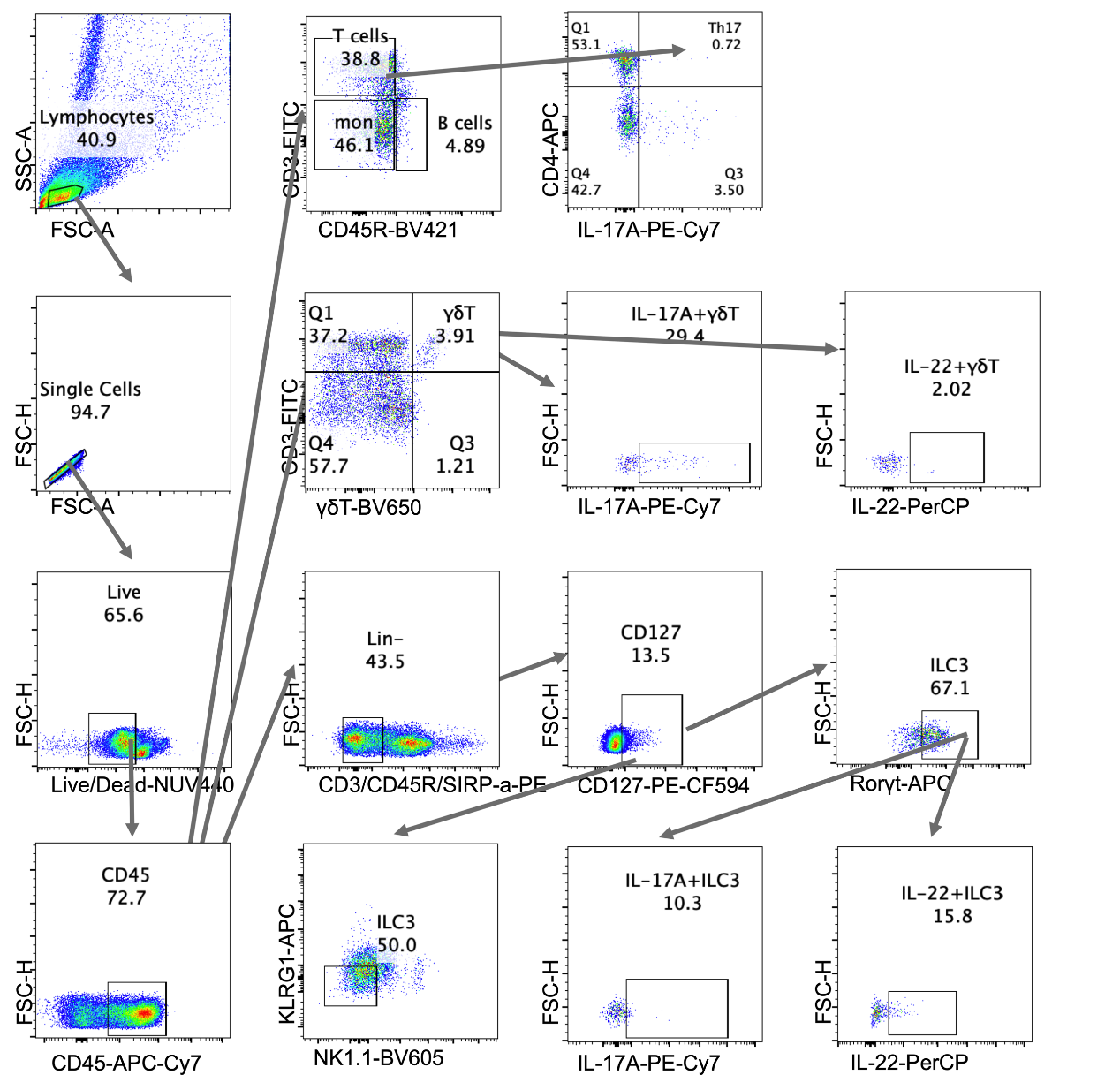
Figure S****1** **FACS gating strategies**

Representative FACS gating strategies used for the identification of different immune cell populations in this study.

**
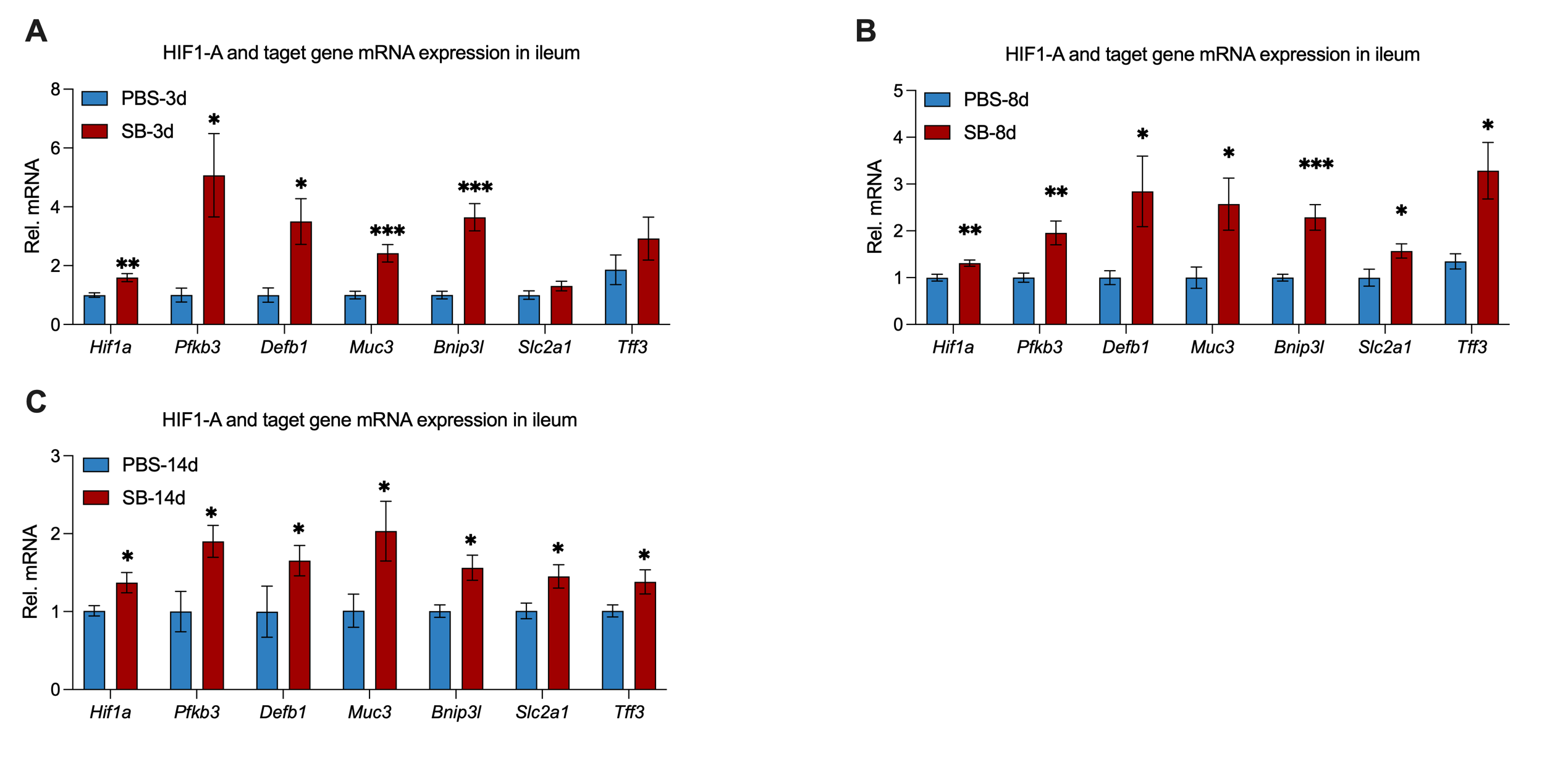
Figure S2 Early administration of *S. boulardii* contributes to intestinal hypoxia in early life**

(A–C) Relative mRNA levels of *Hif1a* and target genes, including *Pfkb3*, *Defb1*, *Muc3*, *Bnip3l*, *Slc2a1*, and *Tff3,* in the rat ileum, n = 8–12.

Data represent mean ± SEM. **P* < 0.05; ***P* < 0.01; ****P* < 0.001. Differences between the two groups were analyzed by two-tailed unpaired Student’s t-test.

**
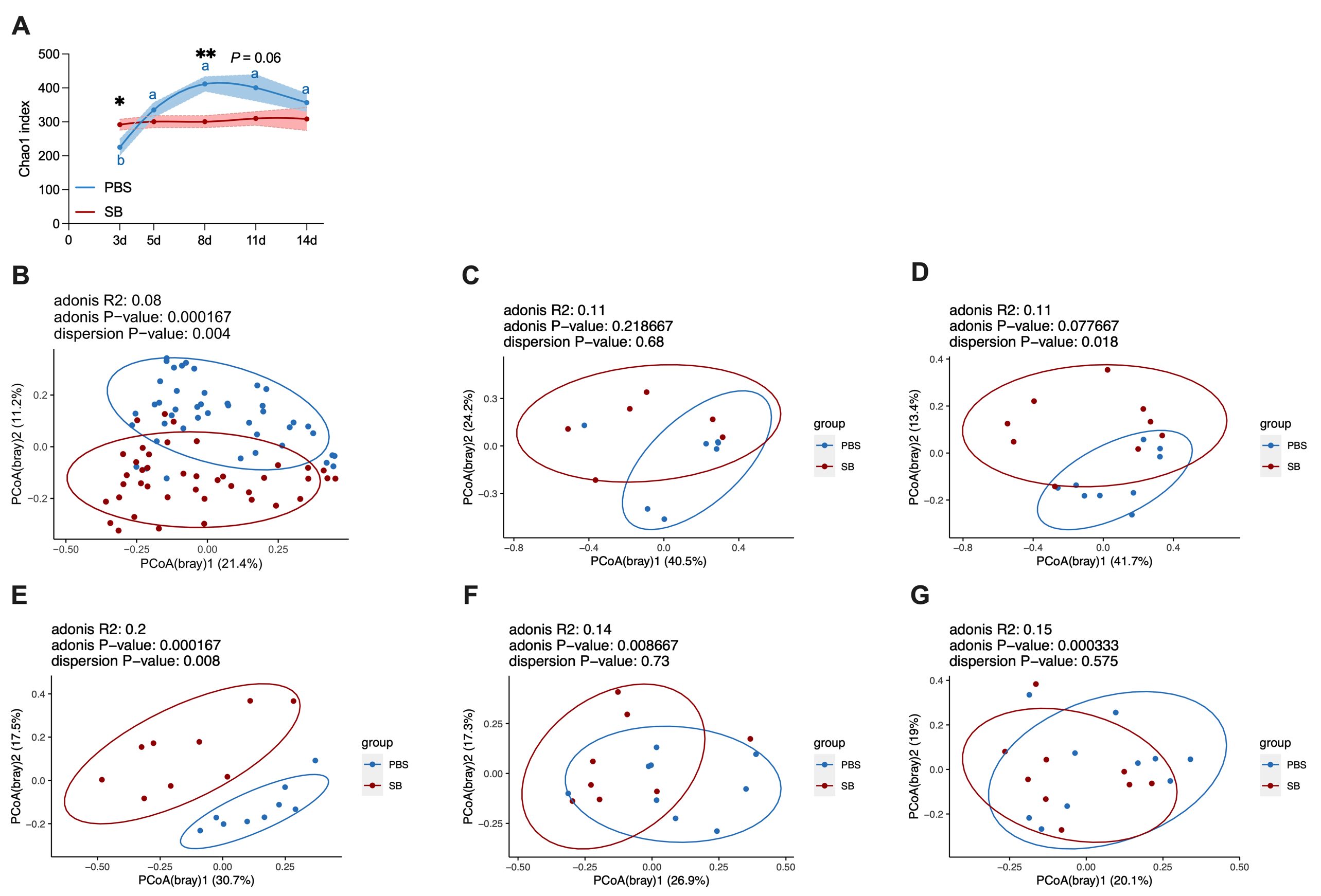
Figure S3 Oral administration of *S. boulardii* changes the composition of the microbiome**

(A) Chao1 index of the ileal microbiome. Different blue letters represent significant differences (*P* < 0.05) between different ages in the PBS group based on one-way ANOVA. The difference between the PBS and SB groups was analyzed by two-tailed unpaired Student’s t test, n = 6–10.

(B) PCoA plot of rats from the PBS and SB groups based on Bray–Curtis analysis. The significance of dissimilarity was calculated by adonis and dispersion analyses, n = 41 and 44.

(C–G) PCoA plot of rats from the PBS and SB groups based on Bray–Curtis analysis at 3, 5, 8, 11, and 14 days. The significance of dissimilarity was calculated by adonis and dispersion analyses, n = 6–10.

**
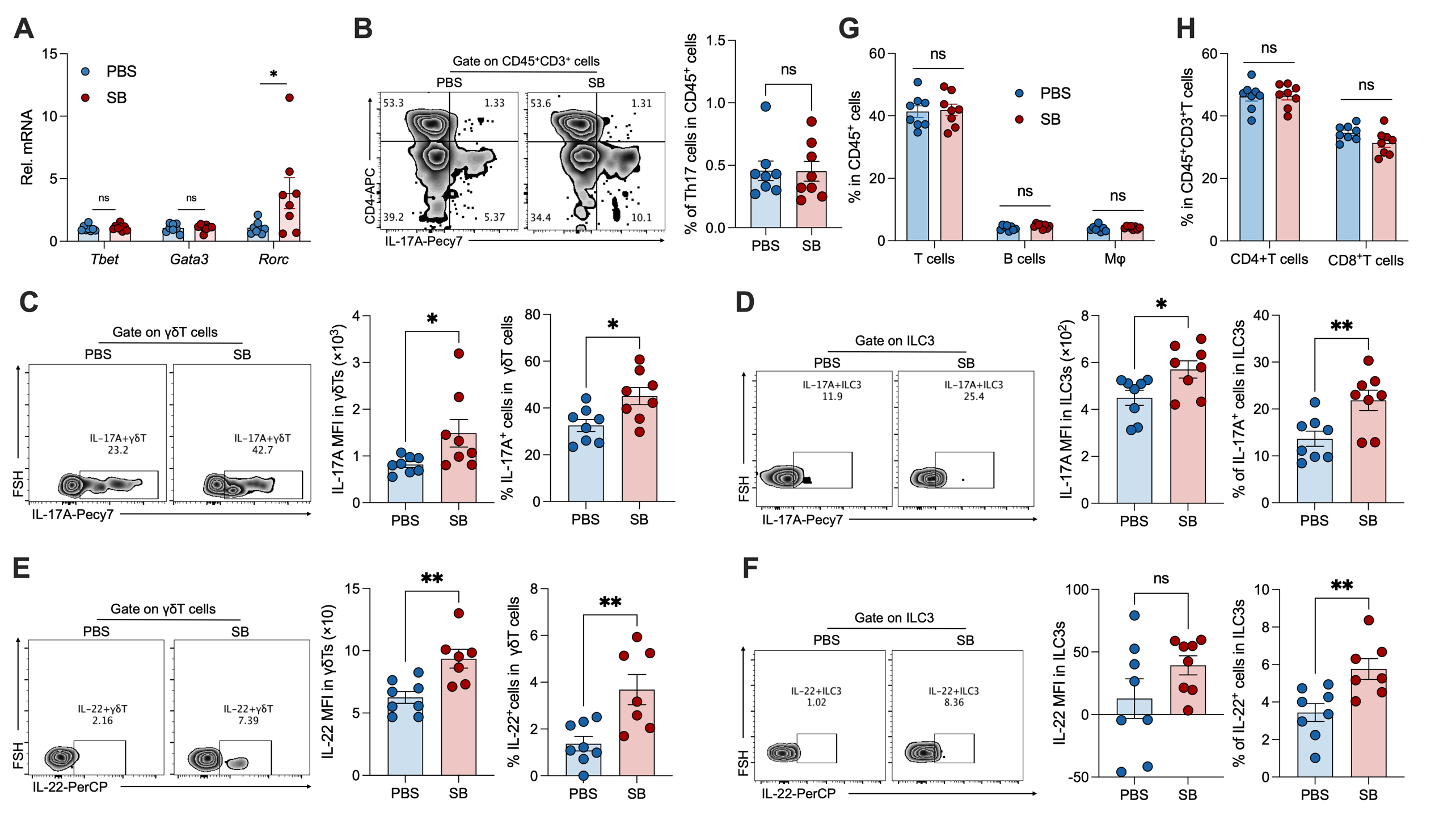
Figure S4 Oral administration of *S. boulardii* facilitates the maturation of type 3 immune cells**

(A) Relative *Tbet*, *Gata3*, and *Rorc* mRNA levels in small intestine LPLs from rats, n = 8.

(B) Representative FACS plots and percentages of Th17 cells in small intestine LPLs from rats, n = 8.

(C–D) Representative FACS plots and percentages of IL-17A^+^ γδT cells and ILC3s in small intestine LPLs from rats, n = 8.

(E–F) Representative FACS plots and percentages of IL-22^+^ γδT cells and ILC3s in small intestine LPLs from rats, n = 8.

(G) FACS analysis of T cells, B cells, and macrophages, n = 8.

(H) FACS analysis of CD4^+^T and CD8^+^T cells, n = 8.

Data represent mean ± SEM. **P* < 0.05; ***P* < 0.01; ns, no significance. Differences between two groups were analyzed by two-tailed unpaired Student’s t-test.

**
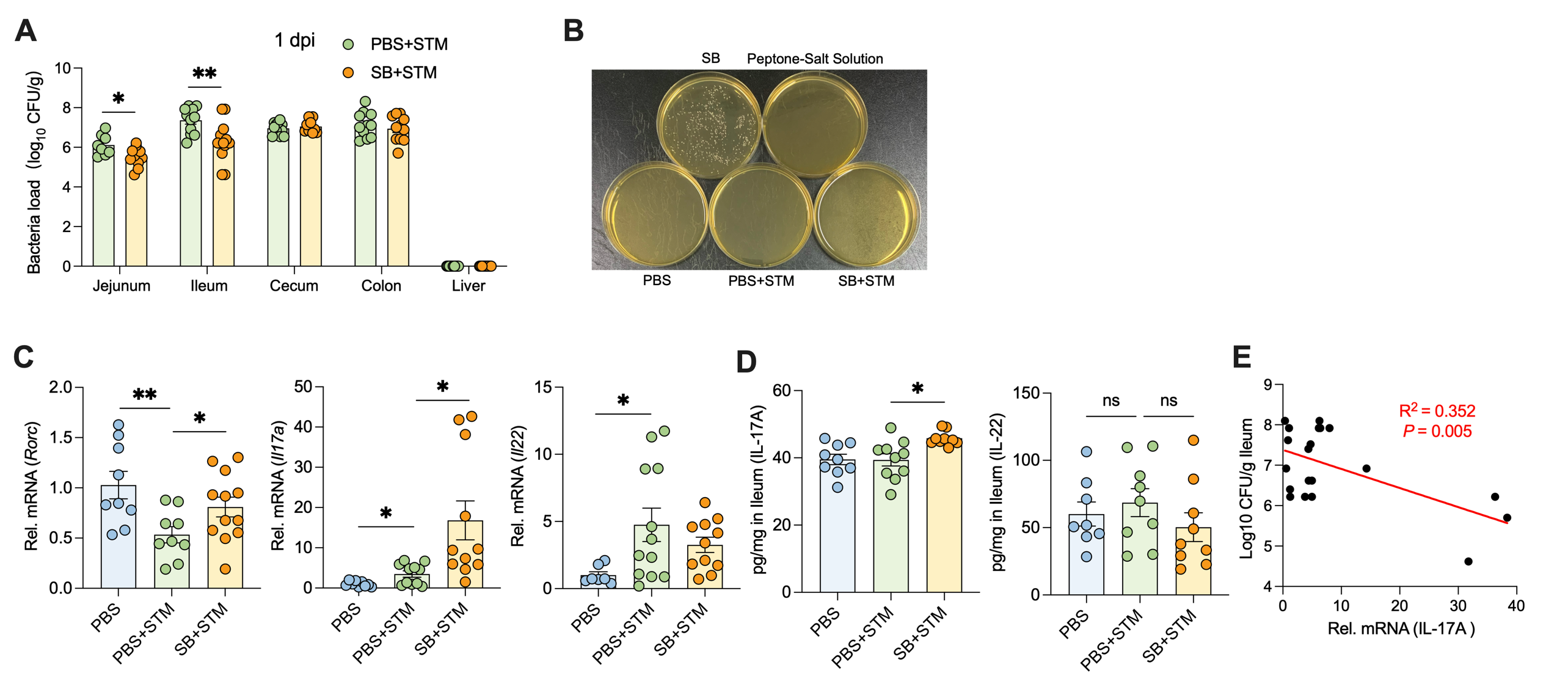
Figure S5 Oral administration of *S. boulardii* confers protection from *S.* Typhimurium infection**

(A) *S.* Typhimurium burden in the jejunum, ileum, cecum, colon, and liver of rats in the PBS, PBS+STM, and SB+STM groups at 1 dpi, n = 10–12.

(B) *S. boulardii* burden in the ileal content at 1 dpi, n = 10–12.

(C) RT-qPCR results of mRNAs encoded by the type 3 immune factors *Rorc*, *Il17a*, *Il22* in the ileum, n = 10–12.

(D) ELISA analysis of IL-17A and IL-22 levels in the rat ileum, n = 10–12.

(E) Correlation between STM burden and *Il17a* mRNA levels in the rat ileum, n = 23.

Data represent mean ± SEM. **P* < 0.05; ***P* < 0.01; ns, no significance. Differences between the two groups were analyzed by two-tailed unpaired Student’s t-test, except for (E).

**
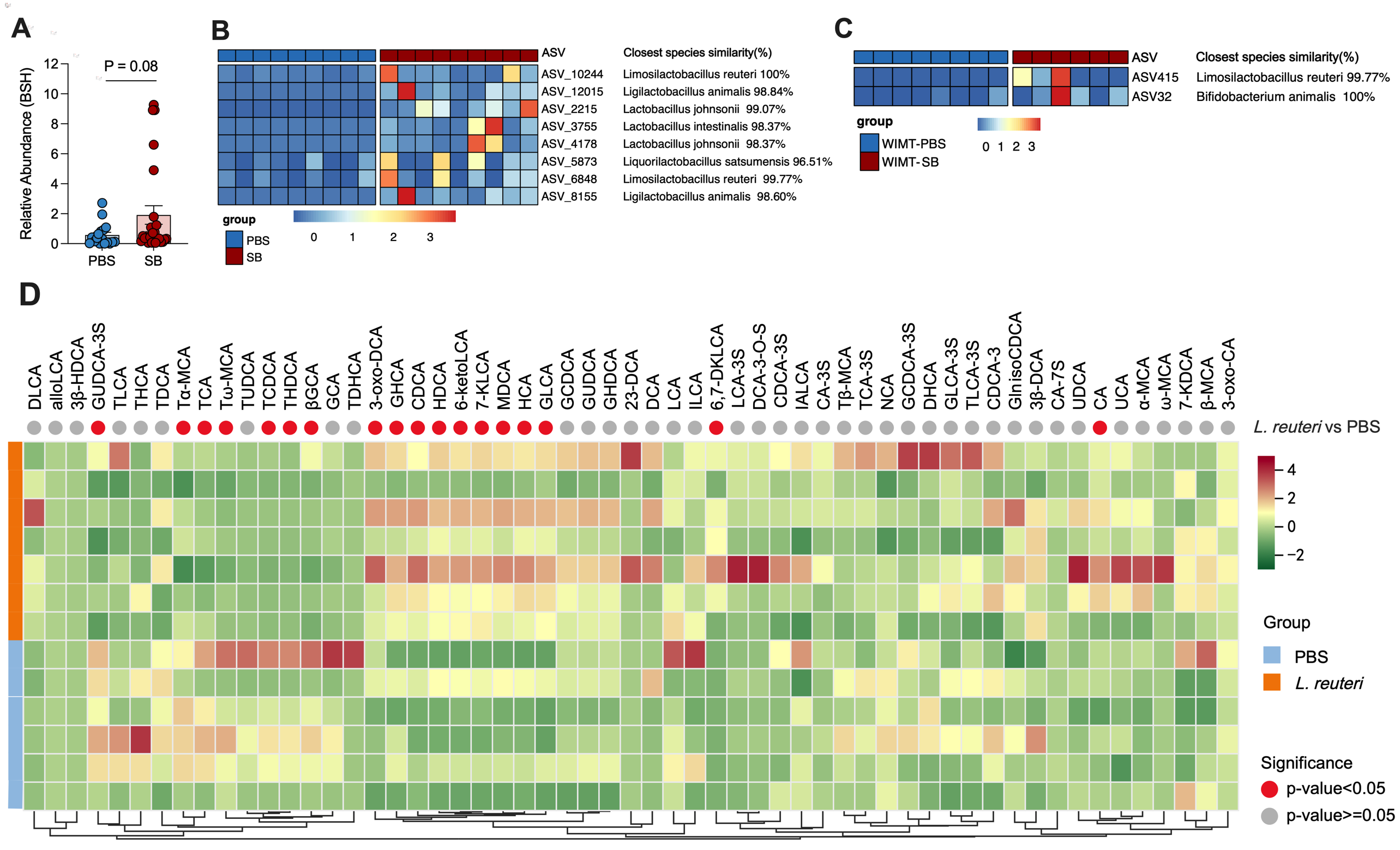
Figure S6 *L. reuteri* altered BA profiles**

(A) RT-qPCR analysis of relative *BSH* abundance in the ileal content of PBS and SB rats, n = 17 and 25.

(B, C) Heatmap showing the significantly different relative expression levels of ASVs between rats in the PBS and SB groups and rats in the WIMT-PBS and WIMT-SB groups. The ASV sequences were compared using EzBioCloud.

(D) Heatmap showing the absolute levels of BAs between the PBS and *L. reuteri* groups. Red dots indicate *P* < 0.05, gray dots indicate *P* ≥ 0.05, n = 6–7.

Data represent mean ± SEM. **P* < 0.05. Differences between the two groups were analyzed by two-tailed unpaired Student’s t-test, except for (A) and (B).

**
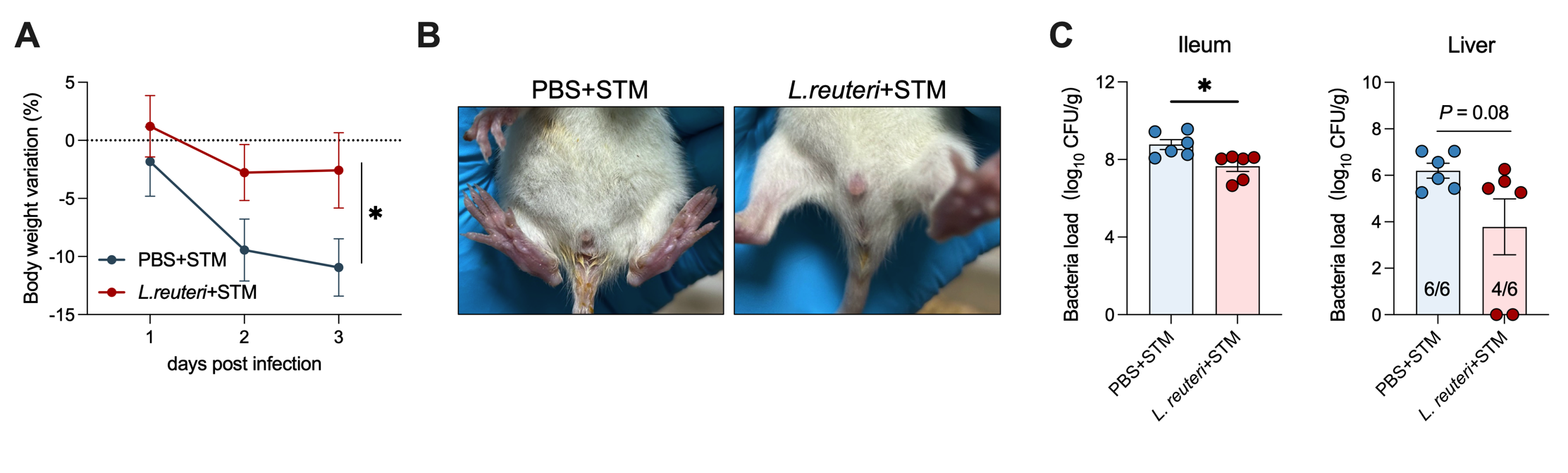
Figure S7 *L. reuteri* protects against *S.* Typhimurium infection**

(A) Bodyweight changes in the PBS + STM and *L. reuteri* + STM groups, n = 6.

(B) Representative diarrhea images of rats, n = 6.

(C) STM burden in the ileum and liver at 3 dpi, n = 6.

Data represent mean ± SEM. **P* < 0.05; ***P* < 0.01; ****P* < 0.001. Differences between the two groups were analyzed by two-tailed unpaired Student’s t-test.


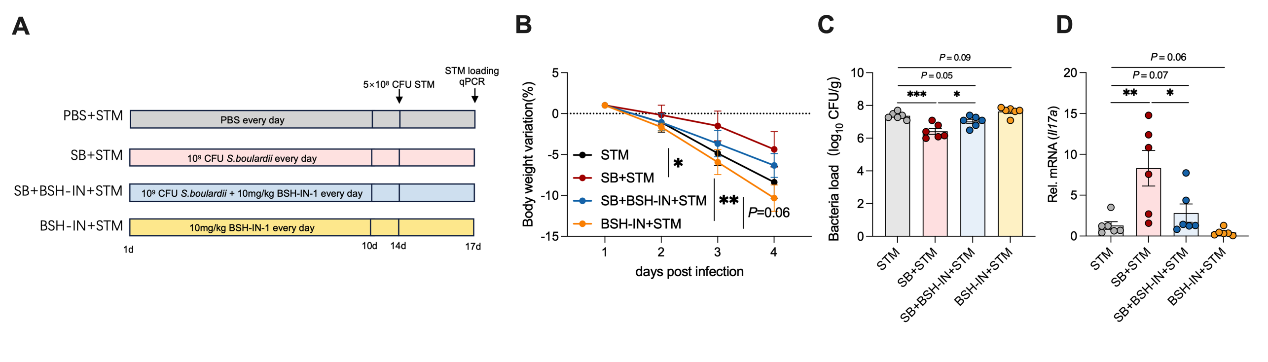
**Figure S8 Impact of BSH inhibition on *S. boulardii*-mediated protection against S*.* Typhimurium infection**

(A) SB and BSH-IN treatment and STM infection experimental timeline.

(B) Bodyweight changes in the PBS + STM, SB + STM, SB + BSH-IN + STM, and BSH-IN + STM groups, n = 7–10.

(C) STM burden in the rat ileum in the PBS + STM, SB + STM, SB + BSH-IN + STM, and BSH-IN + STM groups, n = 6.

(D) RT-qPCR results of *Il17a* in the ileum, n = 6.

Data represent mean ± SEM. **P* < 0.05; ***P* < 0.01; ****P* < 0.001. ns, no significance. Differences between the two groups were analyzed by two-tailed unpaired Student’s t-test.

**
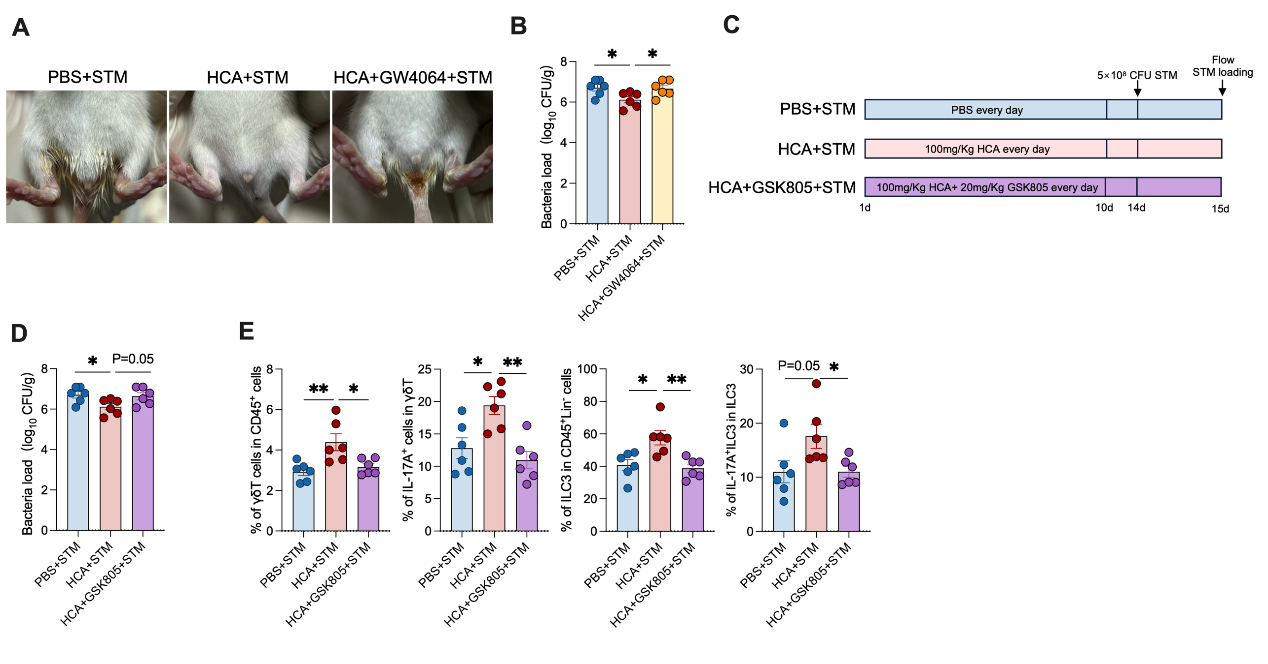
Figure S9 HCA enhances the function of type 3 immune cells via inhibition of FXR**

(A) Representative diarrhea images from the PBS + STM, HCA + STM, and HCA + GW4064 + STM groups.

(B) STM burden in the rat ileum at 1 dpi, n = 6.

(C) HCA and GSK805 treatment and STM infection experimental timeline.

(D) STM burden in the rat ileum in the PBS + STM, HCA + STM, and HCA + GSK805 + STM groups at 1 dpi, n = 6.

(E) Representative FACS plots and percentages of γδT cells, IL-17A^+^γδT cells, ILC3s, and IL-17A^+^ILC3s in small intestine LPLs, n = 6.

Data represent mean ± SEM. **P* < 0.05; ***P* < 0.01; ns, no significance. Differences between the two groups were analyzed by two-tailed unpaired Student’s t-test.

**
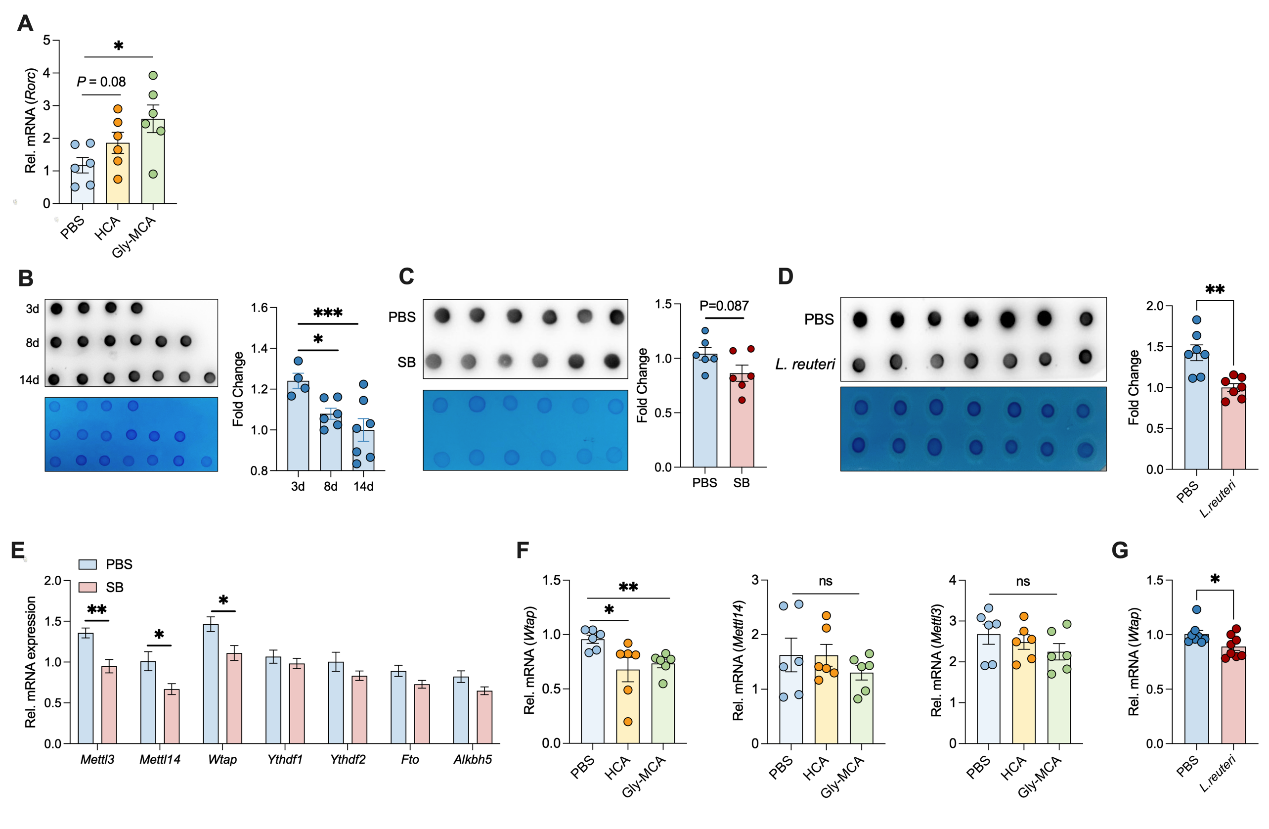
Figure S****10 HCA affects *Rorc* transcription via m^6^A**

(A) RT-qPCR analysis of *Rorc* mRNA expression in LPLs isolated from rats in the PBS, HCA, and Gly-MCA groups, n = 6.

(B) Dot blot analysis of m^6^A levels of mRNA isolated from total RNA in LPLs from 3-, 8-, and 14-day-old rats, n = 4–7.

(C) Dot blot analysis of m^6^A levels of mRNA isolated from total RNA in LPLs from rats in the PBS and SB groups, n = 6.

(D) Dot blot analysis of m^6^A levels of mRNA isolated from total RNA in LPLs from rats in the PBS and *L. reuteri* groups, n = 7.

(E) RT-qPCR analysis of the mRNA levels of m^6^A-related genes *Wtap*, *Mettl3*, *Mettl14*, *Ythdf1*, *Ythdf2*, *Fto*, and *Alkbh5* in LPLs isolated from rats in the PBS and SB groups, n = 8.

(F) RT-qPCR analysis of the mRNA levels of m^6^A-related genes *Wtap*, *Mettl3*, and *Mettl14* in LPLs isolated from rats in the PBS, HCA, and Gly-MCA groups, n = 8.

1. RT-qPCR analysis of *Wtap* mRNA levels in LPLs from rats in the PBS and L. *reuteri* groups, n = 8.

Data represent mean ± SEM. **P* < 0.05; ***P* < 0.01; ****P* < 0.001; ns, no significance. Differences between the two groups were analyzed by two-tailed unpaired Student’s t-test.
